# Green Micelle-Enhanced Spectrofluorimetric Determination of Gefitinib in Pharmaceutical Tablets Using Cremophor RH 40: Response-Surface Optimization and Time-Dependent Density Functional Theory (TD-DFT) Interpretation

**DOI:** 10.3390/ijms27209015

**Published:** 2026-10-11

**Authors:** Ahmed H. Bakheit, Abdullah M. A. Alzaid, Abdulrahman A. Almajed

**Affiliations:** Department of Pharmaceutical Chemistry, College of Pharmacy, King Saud University, Riyadh 11451, Saudi Arabia; a.al_zaid@hotmail.com (A.M.A.A.); aalmajed@ksu.edu.sa (A.A.A.)

**Keywords:** gefitinib, Cremophor RH 40, micelle-enhanced spectrofluorimetry, green analytical chemistry, TD-DFT, pharmaceutical analysis

## Abstract

Gefitinib is an epidermal growth factor receptor inhibitor used in non-small-cell lung cancer. This study developed a micelle-enhanced spectrofluorimetric method for its determination in tablets using native fluorescence and Cremophor RH 40 without derivatization. Experimental conditions were screened by a univariate approach and further evaluated by response-surface methodology. The quadratic model was significant (F = 83.26, *p* = 0.0019), and three confirmation experiments yielded prediction errors of 0.515–0.954%, supporting local agreement at the selected conditions. Fluorescence intensity was measured at an emission wavelength of 394 nm following excitation at 333 nm. The method was linear over 50–1200 ng mL^−1^ (*r* = 0.9997). The calculated limits of detection and quantification were 6.89 and 20.89 ng mL^−1^, respectively, while 50 ng mL^−1^ was retained as the lowest validated concentration. The method showed acceptable accuracy, repeatability, and selectivity for the investigated tablet matrix. Robustness testing indicated that pH requires careful control around 8.0. Analysis of pooled commercial tablets yielded an assay of 100.21 ± 1.86% of the label claim. TD-DFT calculations supported qualitative interpretation of the electronic transitions. The AGREE score was 0.61, indicating a more favorable environmental profile than the reference spectrofluorimetric method comparator (0.46).

## 1. Introduction

On 5 May 2003, gefitinib (Iressa^®^; ZD1839), a first-generation epidermal growth factor receptor (EGFR) tyrosine kinase inhibitor, received accelerated approval from the United States Food and Drug Administration (FDA) for the treatment of locally advanced or metastatic non-small-cell lung cancer (NSCLC) in patients who had failed both platinum-based and docetaxel chemotherapy. Gefitinib acts by competitively binding to the ATP-binding site of EGFR, inhibiting receptor autophosphorylation and thereby suppressing tumor cell proliferation [1,2].

**Figure 1:**
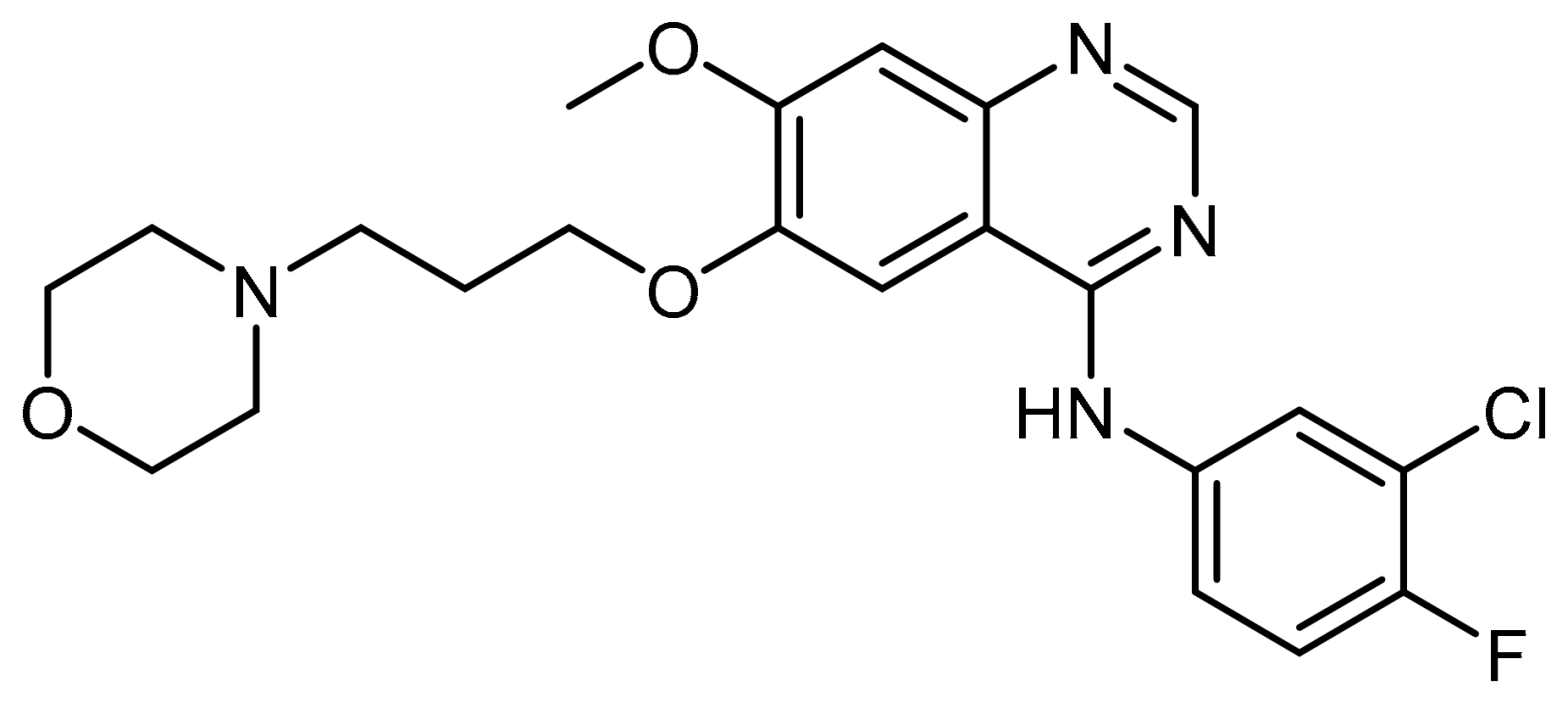
Chemical structure of gefitinib (GFB).

The chemical structure of GFB is N-(3-chloro-4-fluorophenyl)-7-methoxy-6-(3-morpholin-4-ylpropoxy)quinazolin-4-amine (Figure 1), with a molecular weight of 446.90 g/mol for the free base. In addition to its established use in NSCLC, gefitinib has been investigated in other oncological settings, including breast cancer, and reported to be tolerable in the cited study in patients with recurrent endometrial, ovarian, or primary peritoneal carcinoma [3]. Preclinical evidence further indicates that GFB can induce PINK1/Parkin-mediated mitophagy through optineurin (OPTN), pointing to a possible therapeutic relevance in neurodegenerative diseases characterized by mitophagy dysfunction [4].

A variety of analytical methods have been reported for GFB quantification, including ultraviolet (UV) spectrophotometry [5,6], Spectrofluorometry [7], high-performance liquid chromatography (HPLC) [7,8,9], liquid chromatography–mass spectrometry (LC-MS) [10,11], ultra-performance liquid chromatography (UPLC) [12], and high-performance thin-layer chromatography (HPTLC) [13]. A literature survey revealed that only one spectrofluorimetric method has previously been reported for the determination of gefitinib in bulk drug and pharmaceutical dosage forms by Ramesh and Senthil Kumar. However, this reported method utilized ethanol as the analytical medium and was primarily developed for conventional pharmaceutical analysis. In contrast, the present method was designed according to the principles of green analytical chemistry, employing environmentally benign experimental conditions with reduced solvent consumption and lower environmental impact. Furthermore, as detailed in Section 2.5, the proposed method provides lower limits of detection and quantification than the reported procedure. Its greenness advantage, however, is confined to the final measurement step: the reported method uses ethanol, a solvent generally regarded as greener than the acetonitrile used here for stock and sample preparation, and this is discussed quantitatively in Section 2.6. Spectrophotometric methods, while simple and cost-effective, generally lack the selectivity and sensitivity required for trace-level analysis. Conversely, chromatographic methods provide high sensitivity and selectivity but are often labor-intensive, time-consuming, and dependent on expensive instrumentation and specialized personnel. Therefore, the proposed green spectrofluorimetric method offers an attractive alternative for routine quality-control applications by combining simplicity, environmental sustainability, cost-effectiveness, and enhanced sensitivity. Spectrofluorimetry is well recognized for combining high sensitivity with procedural simplicity. Micelle-enhanced spectrofluorimetry, in particular, has emerged as an effective way of boosting the fluorescence quantum yield of weakly fluorescent drugs by incorporating them into the hydrophobic core of surfactant micelles, without using organic solvents, making the approach both sensitive and environmentally friendly [14]. Non-ionic and anionic surfactants such as sodium dodecyl sulfate (SDS) [15,16], polysorbates (Tweens) [17,18], and cyclodextrins [14] have all been employed for this purpose.

The present work must also be positioned with respect to earlier micellar spectrofluorimetric assays that used the same class of polyoxyethylated castor-oil surfactant, because the underlying concept of micellar fluorescence enhancement is not new. Cremophor (Kolliphor) RH 40 has previously been evaluated as a fluorescence enhancer for several tyrosine kinase inhibitors, most directly in a micellar-based spectrofluorimetric determination of lapatinib in tablets and urine [19]. That study established the general feasibility of the surfactant and refined the medium by response-surface methodology, but it operated at a final surfactant concentration of about 0.8% *w*/*v* in strongly alkaline phosphate buffer (pH 12), reported a linear range of 50–1000 ng mL^−1^ with limits of detection and quantification of 27.31 and 82.75 ng mL^−1^, described its computational component in terms of response-surface modelling and pH-dependent microspecies distribution rather than quantum-chemical calculation, and assessed greenness only qualitatively. The present study differs in analyte and in five substantive respects. (i) To the best of our knowledge, this is the first micelle-enhanced spectrofluorimetric assay for gefitinib, a drug whose fluorophore, protonation behaviour and lipophilicity differ from those of lapatinib, so the optimum micellar conditions could not be transferred and had to be established de novo. (ii) The optimized medium contains 0.005% *w*/*v* Cremophor RH 40, approximately 160-fold less surfactant, at pH 8 instead of pH 12, which avoids strongly alkaline conditions and the associated risk of base-mediated degradation of the analyte and of the surfactant esters. (iii) The limits of detection and quantification are approximately four-fold lower. (iv) The photophysical basis of the enhancement is examined by singlet and triplet TD-DFT calculations of the frontier molecular orbitals and of the excitation and emission transitions in six media, which has not previously been reported for a Cremophor RH 40 micellar assay. (v) The environmental profile is quantified with the AGREE metric against a defined comparator rather than asserted. The novelty claimed here therefore lies in the analyte, in the markedly milder and more reagent-economical micellar conditions, in the quantum-chemical rationalization of the solvent- and micelle-dependent fluorescence, and in the quantitative greenness assessment, and not in the use of a micellar medium as such.

This study develops a simple, rapid, micelle-enhanced spectrofluorimetric method for the quantification of gefitinib in pharmaceutical tablets, exploiting its intrinsic (native) fluorescence and thereby avoiding derivatization. A two-tier optimization strategy was adopted. The type of surfactant system, Cremophor RH 40 concentration, pH, buffer volume, and diluting solvent were first screened by a classical univariate (“one-factor-at-a-time (OFAT)”) approach to identify the promising region of the experimental domain and to eliminate poorly performing surfactants (Section 2.2.1, Section 2.2.2, Section 2.2.3, Section 2.2.4 and Section 2.2.5). The three factors expected to interact most strongly—buffer volume, Cremophor RH 40 concentration, and pH—were then re-examined within a narrower, OFAT-informed range using a Box–Behnken response-surface design (RSM/BBD) to test for curvature and factor interactions and to obtain a statistically defensible optimum (Section 2.2.6). In parallel, the electronic origin of the native fluorescence of GFB, and its marked solvent dependence, were examined by density functional theory (DFT) and TD-DFT calculations of the frontier molecular orbitals and of the excitation and emission spectra in six representative media. Based on the current literature search, this is the first spectrofluorimetric method reported for gefitinib that combines univariate screening with response-surface methodology (RSM) confirmation and a DFT-based mechanistic rationale for this drug.

ICH Q2(R2) links validation to the intended purpose of the analytical procedure, while Q14 introduces an analytical target profile as a prospective description of what the analytical procedure is expected to measure and the required performance.

## 2. Results & Discussion

Spectrofluorimetric analysis combines high sensitivity with operational simplicity over a wide concentration range and was evaluated for assay applications suited to the assay of gefitinib in its dosage form. In this work, sensitivity was further enhanced by the addition of a surface-active agent that promotes micelle formation. Several micelle-enhanced spectrofluorimetric methods for structurally related tyrosine kinase inhibitors were previously reported [20,21,22,23,24]; building on that experience, and given the well-established ability of micelles to increase the RFI of poorly fluorescent drugs. The present assay for GFB was optimized with minimal organic solvent in the final mixture. The two-tier optimization strategy, together with its scientific rationale, has been established in the Introduction and is not restated in this section. The sequential screening experiments and confirmatory response-surface analysis are reported in detail in Section 2.2.1, Section 2.2.2, Section 2.2.3, Section 2.2.4, Section 2.2.5 and Section 2.2.6.

### 2.1. Computational Analysis of GFB

#### 2.1.1. Singlet–Triplet TD-DFT Electrostatic Potential Analysis of Gefitinib

**Figure 2:**
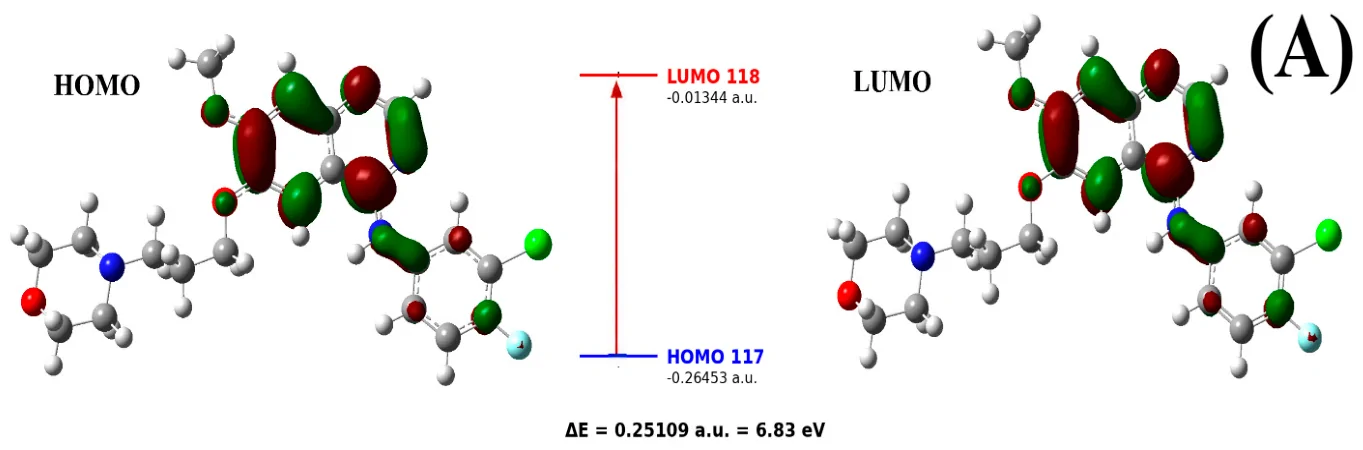

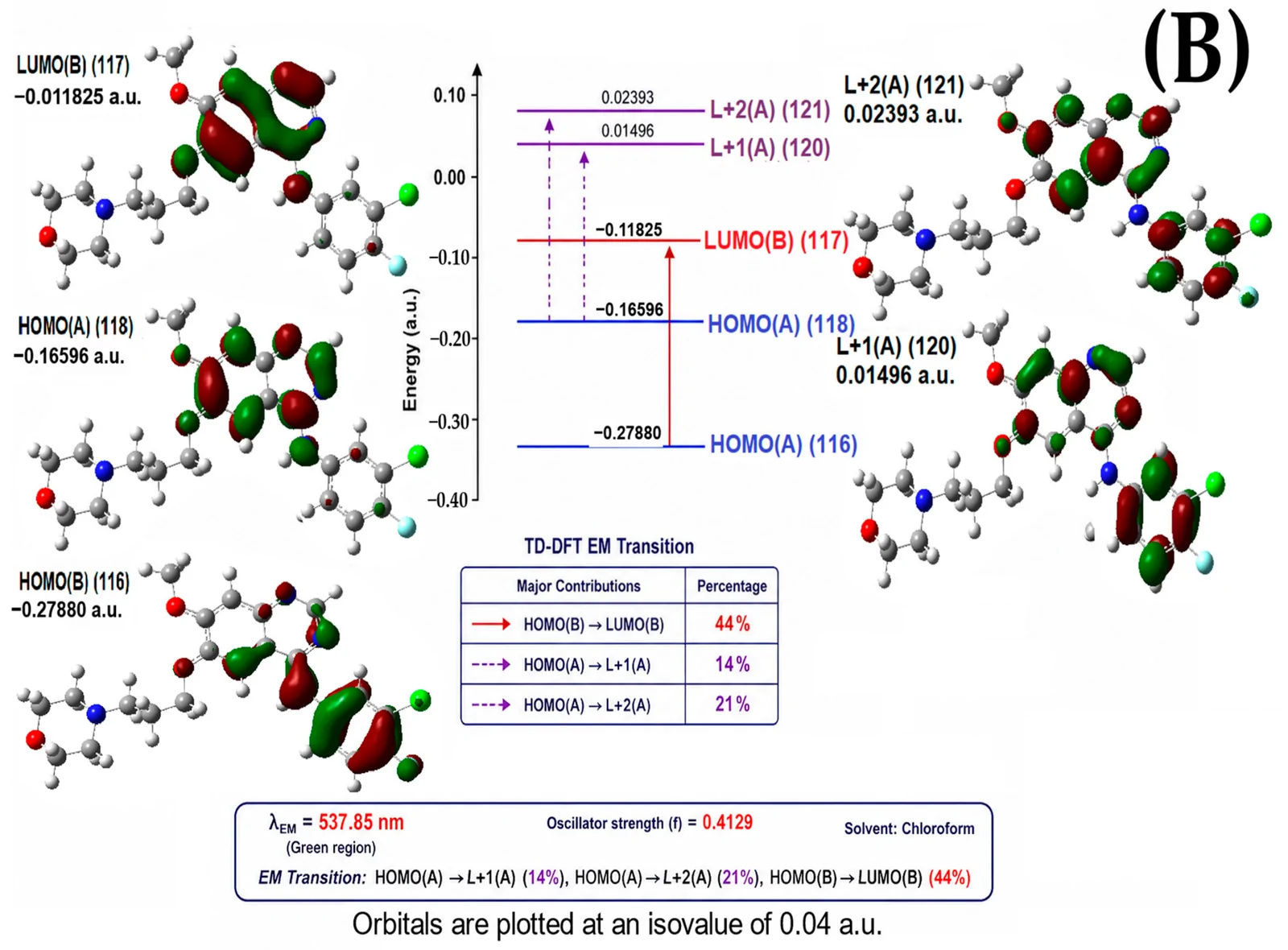
Frontier molecular orbital (FMO) diagrams of gefitinib in the chloroform phase. (**A**) HOMO and LUMO involved in the EX transition, with a HOMO–LUMO energy gap of 0.25109 a.u. (6.83 eV). (**B**) Frontier molecular orbitals involved in the EM transition, showing the dominant HOMO(B) → LUMO(B) (44%), HOMO(A) → L + 2(A) (21%), and HOMO(A) → L + 1(A) (14%) electronic transitions at 537.85 nm.

The photophysical response of gefitinib (GFB) was evaluated by combining experimental fluorescence measurements in different media with singlet–triplet TD-DFT calculations. The experimental excitation maxima of GFB in conventional solvents were located within a narrow ultraviolet region, appearing at 286 nm in chloroform, 282–283 nm in acetonitrile, 284 nm in ethanol, 288 nm in methanol, and 290 nm in water. This limited variation indicates that the ground-state absorption/excitation process is only moderately influenced by the bulk solvent. This observation agrees with the theoretical singlet TD-DFT results, where the principal excitation band was calculated close to 292 nm in solvated media and at 286.60 nm in the gas phase. The major calculated excitation transition was assigned predominantly to HOMO (117) → LUMO (118), with about 89% contribution in chloroform and acetonitrile and high oscillator strengths of 0.6669 and 0.6535 (Figure 2A and Table S1), respectively. Therefore, the main excitation of GFB can be attributed to an allowed singlet frontier molecular orbital transition, most likely with π→π* character, involving redistribution of electron density from the occupied molecular orbital region toward the corresponding unoccupied orbital [25].

Unlike the excitation process, the experimental fluorescence emission showed strong medium dependence. The most intense fluorescence response was obtained in chloroform, where GFB exhibited λ_EM_ = 488 nm with an RFI of 7547.22. Acetonitrile also produced a long-wavelength emission band at 496 nm, with an RFI of 1744.25. In contrast, protic solvents produced weaker and blue-shifted emission, with λ_EM_ = 418 nm in methanol, 412 nm in ethanol, and 444 nm in water (Tables S1 and S2). These results indicate that the emissive state of GFB is more sensitive than the absorptive state to solvent polarity, hydrogen-bonding capacity, local solvation, and non-radiative deactivation pathways. The weak emission observed in water is consistent with aqueous quenching and poor protection of the excited fluorophore, whereas the strong fluorescence in chloroform suggests that a less quenching environment favors radiative relaxation [26,27].

The triplet-related TD-DFT emission calculations provided further insight into the relaxed excited-state electronic structure. In the gas phase, the main calculated emission-related transition was observed at 519.41 nm, with an oscillator strength of 0.3485. This transition was mainly assigned to HOMO(B) → LUMO(B) (47%), together with additional α-spin contributions from HOMO(A) → L + 1(A) (17%) and HOMO(A) → L + 2(A) (15%) (Figure 2B and Table 1 and Table S1). In solvated media, the calculated emission-related transitions were red-shifted to approximately 534–538 nm, including 537.85 nm in chloroform, 534.88 nm in acetonitrile, 535.44 nm in ethanol, 534.39 nm in methanol, and 534.51 nm in water (Table 1). This red shift relative to the gas phase suggests stabilization of the relaxed triplet-related electronic configuration by the solvent field. However, the calculated emission wavelengths are longer than the experimental fluorescence maxima because the theoretical emission-related transitions were obtained from triplet-spin electronic structures, whereas the experimentally measured fluorescence is mainly associated with singlet-state radiative emission. Thus, the calculated EM values should be interpreted as triplet-related descriptors of excited-state relaxation rather than direct predictions of experimental fluorescence maxima [27,28]. The magnitude of this discrepancy should be stated explicitly, because it defines the limits of what the calculations can support. Relative to the measured fluorescence maxima, the calculated triplet-derived emission wavelengths are longer by 39 nm (0.18 eV) in acetonitrile, 50 nm (0.24 eV) in chloroform, 91 nm (0.47 eV) in water, 116 nm (0.65 eV) in methanol and 123 nm (0.69 eV) in ethanol. More importantly, the micellar emission maximum at 394 nm lies entirely outside the range of all calculated values, since no explicit or implicit representation of the Cremophor RH 40 pseudophase was included and a continuum solvation model cannot reproduce the microheterogeneous, motionally restricted environment experienced by GFB at the micelle–water interface. The TD-DFT results are therefore used in this work only to identify which orbitals and which type of transition are involved; they are not used, and cannot be used, to reproduce, predict or validate the fluorescence emission wavelengths, quantum yields or intensities of the micellar system.

**Figure 3:**
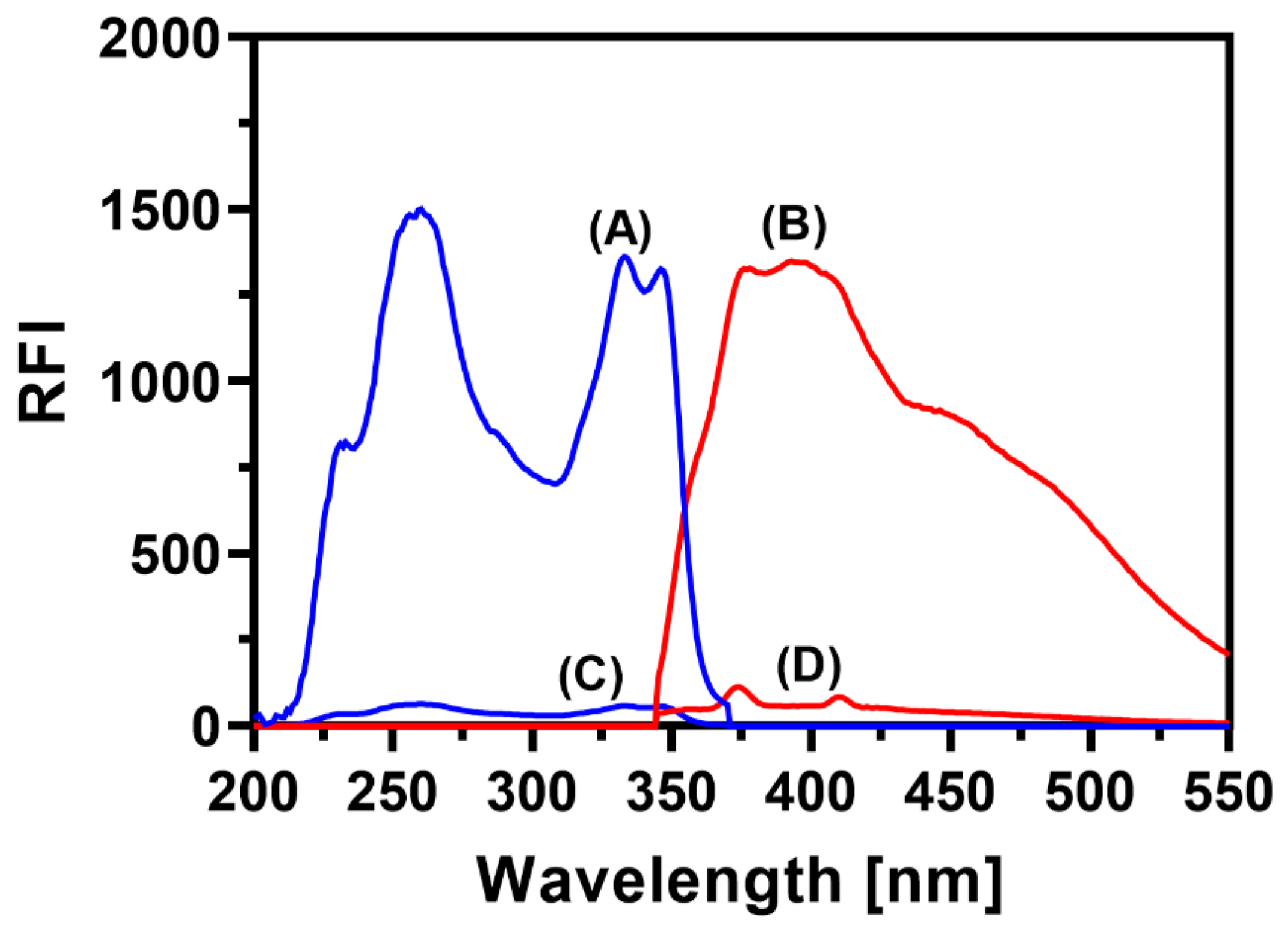
Excitation spectra (blue lines) and emission spectra (red lines) of GFB (500 ng/mL) in 0.005% *w*/*v* Cremophor RH 40 (**A**,**B**) compared with GFB (500 ng/mL) in plain aqueous solution (**C**,**D**); the excitation and emission spectra of 0.005% *w*/*v* Cremophor RH 40 alone (blank) are overlaid for reference.

The Cremophor RH 40 micellar medium produced a distinct spectral behavior compared with conventional solvents. Under the optimized micellar conditions, the selected wavelengths were λ_EX_ = 333 nm and λ_EM_ = 394 nm (Figure 3) & (Table 1). The excitation maximum at 333 nm is markedly red-shifted relative to the excitation maxima in conventional solvents (282–290 nm) and the calculated singlet TD-DFT band near 292 nm. This shift suggests that GFB is not present as a freely solvated molecule in bulk aqueous buffer, but is instead incorporated into the micellar pseudophase of Cremophor RH 40. Cremophor RH 40 is a nonionic solubilizer/emulsifier composed of hydrophobic fatty ester regions and hydrophilic polyethylene glycol/glycerol ethoxylate domains; therefore, hydrophobic or moderately hydrophobic drug molecules can be accommodated within micellar or interfacial domains rather than remaining fully exposed to bulk water [29,30].

The selection of λ_EM_ = 394 nm is supported by the shape of the micellar fluorescence spectrum. The emission spectrum shows a dominant band around 390–400 nm, together with a broad shoulder extending beyond 450 nm. This broad and asymmetric profile suggests that the recorded micellar emission is likely a composite signal rather than a single isolated fluorescence band. The dominant band at 394 nm can be attributed to micellar-enhanced GFB fluorescence from molecules located in a protected micellar or interfacial microenvironment. The longer-wavelength shoulder may arise from overlapping contributions, including residual GFB emission from another micellar domain, a less restricted GFB population, weak surfactant/buffer background, or scattering effects. Such behavior is reasonable because organized micellar and polymeric media can create microheterogeneous environments with different micropolarity and microviscosity, which can alter fluorescence intensity, wavelength, and band shape [27,28].

Accordingly, λ_EX_ = 333 nm and λ_EM_ = 394 nm were selected as the optimum analytical wavelengths because this wavelength pair provided the strongest and most reproducible micellar-enhanced fluorescence response. Although the shoulder above 450 nm may contain useful photophysical information, this region is less suitable for quantitative measurement because of spectral overlap and possible matrix contribution. The peak at 394 nm provides a clearer analytical maximum and minimizes interference from the broad long-wavelength tail. Similar micellar-enhanced fluorescence behavior has been reported for drug analysis using Cremophor RH 40, where the surfactant distinctly enhanced the intrinsic fluorescence of the analyte in aqueous medium [27,28].

#### 2.1.2. Fluorescence Spectra and Characteristics of GFB

The fluorescence of gefitinib was strongly dependent on solvent polarity and hydrogen-bonding capacity (Table 1). Chloroform produced the highest fluorescence intensity under the tested conditions, consistent with its low polarity and negligible hydrogen-bond-donating ability, which minimizes solvent-induced quenching and favors radiative decay of the excited molecule. Water and the lower alcohols markedly reduced fluorescence intensity, most plausibly because hydrogen-bonding interactions with the quinazoline nitrogen atoms and the anilino NH promote non-radiative (vibrational/collisional) relaxation of the excited state.

The pronounced fluorescence enhancement observed in 0.005% *w*/*v* Cremophor RH 40 at pH 8 (Table 1) may arise from micellar solubilization: Cremophor RH 40 forms hydrophobic domains that can incorporate the lipophilic GFB molecule and shield it from the bulk aqueous phase, thereby suppressing water-mediated non-radiative decay. At pH 8, the morpholinopropoxy side-chain nitrogen of GFB is expected to have a higher deprotonated fraction, increasing the overall hydrophobicity of the molecule and favoring its partitioning into the micellar phase. The hypsochromic and bathochromic shifts in excitation and emission wavelengths on moving from bulk organic solvent to the micellar system (Table 1) reflect the different polarity, rigidity, and hydrogen-bonding environment experienced by GFB at or near the micelle–water interface, as opposed to a homogeneous organic solvent. Chloroform and Cremophor RH 40 therefore enhance GFB fluorescence through the same general principle—exclusion of the fluorophore from a quenching aqueous environment—but the micellar system produces a distinct excitation/emission profile because it provides an interfacial, rather than purely organic, microenvironment; importantly, the micellar system is compatible with an entirely aqueous, green assay.

Figure 3 compares the excitation/emission behavior of GFB before and after micellization. In plain aqueous solution (Figure 3C,D), GFB exhibited an excitation maximum at 333 nm and a low-intensity emission maximum at 394 nm in plain aqueous solution, reflecting the weak native fluorescence of the drug in the absence of micellar protection. Upon addition of the optimum surfactant (Cremophor RH 40) together with pH 8 borate buffer, RFI increases sharply (Figure 3A,B), confirming that micellar encapsulation, rather than a change in the electronic transition itself, is responsible for the sensitivity gain exploited by this method.

**Figure 4:**
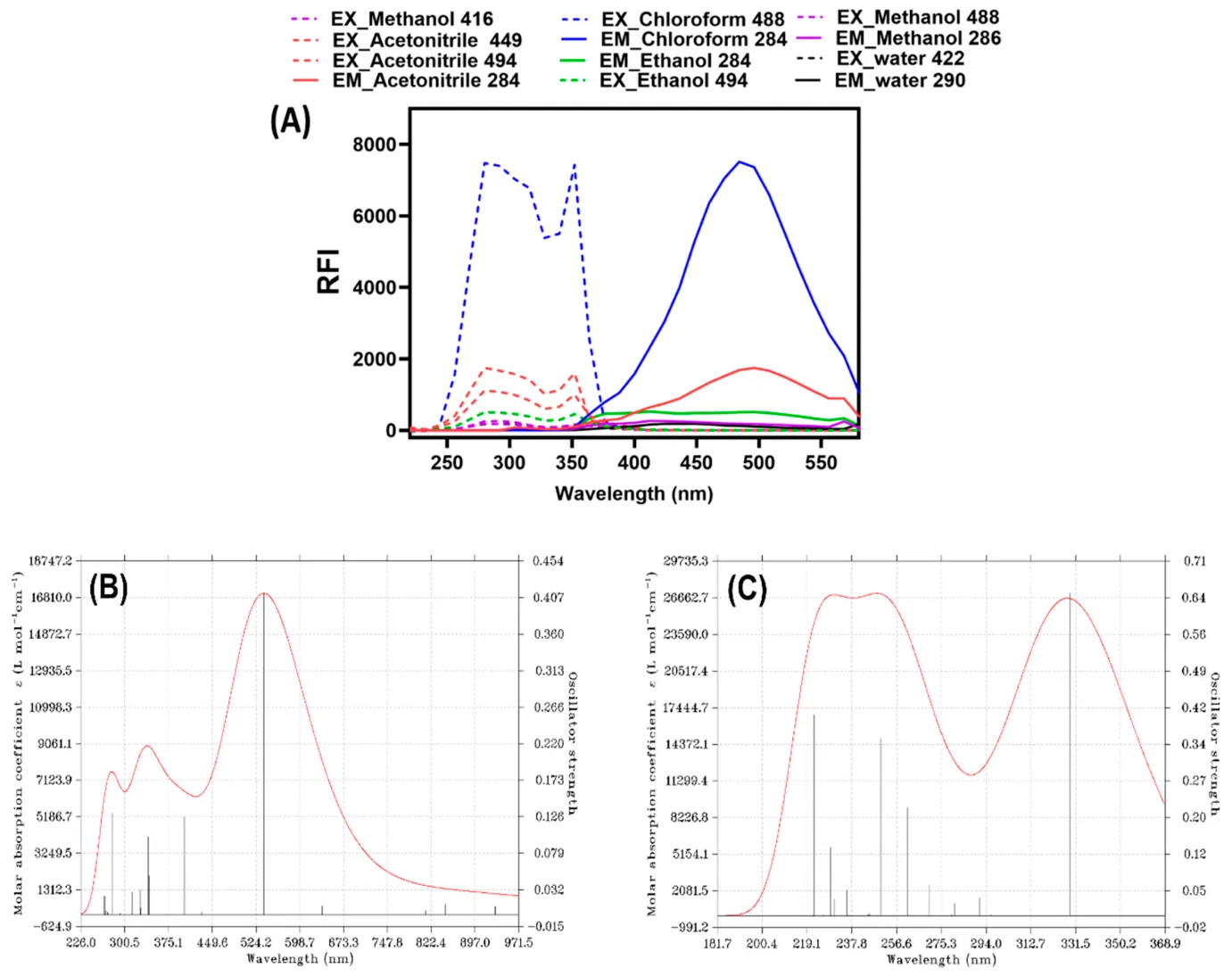
Experimental and predicted spectral profiles of gefitinib. (**A**) Experimental fluorescence spectra recorded in different solvents, presented as RFI versus wavelength; (**B**,**C**) TD-DFT–predicted excitation and emission spectra of gefitinib in chloroform, expressed as molar absorption coefficient, ε (L·mol^−1^·cm^−1^), with oscillator strength shown on the secondary axis. The corresponding predicted spectra in water, acetonitrile, methanol, ethanol, and the gas phase are provided in Supplementary Figure S1 (excitation) and Figure S2 (emission).

To test whether the solvent-dependent fluorescence trend in Table 1 has a rigorous computational basis, TD-DFT excitation and emission spectra of GFB were additionally predicted in five further media—water, acetonitrile, methanol, ethanol, and the gas phase—alongside the chloroform case already shown in Figure 4B,C, Figures S1 and S2. Presenting all six media in parallel allows the computed spectral envelopes to be compared directly against the experimental ranking in Table 1 (chloroform ≫ acetonitrile > ethanol > methanol > water). Qualitatively, low-polarity, non-hydrogen-bonding media are predicted to give the most red-shifted transitions with larger calculated oscillator strengths, consistent with the substantially higher experimental RFI measured in chloroform than in the protic or strongly hydrogen-bonding solvents (Table 1); the gas-phase spectrum provides the reference unperturbed transition energy (Section 3.1) against which all solvated predictions can be benchmarked.

#### 2.1.3. Solvent Polarity, Dipole Moment, and Solubility Behavior of GFB

The solvent effect on GFB fluorescence was discussed using the dielectric properties of chloroform, acetonitrile, ethanol, methanol, and water. Their static dielectric constants increase in the order: chloroform (ε_s_ = 4.7), ethanol (24.3), methanol (33.0), acetonitrile (36.0), and water (78.4). Higher dielectric solvents can better stabilize polar electronic states of GFB; however, solvent polarity alone does not fully explain GFB behavior because solubility, hydrogen bonding, dispersion, and micellar partitioning also contribute [31]. The calculated dipole moments of GFB in the gas phase and in the investigated solvents, together with the corresponding dielectric constants, are summarized in Table 2.

Chloroform may favor solubilization of the hydrophobic aromatic regions of GFB but provides limited electrostatic stabilization. Acetonitrile, as a polar aprotic solvent, offers strong dipolar stabilization, while ethanol and methanol provide both polarity and hydrogen-bonding ability. Water has the highest dielectric constant, but the poor intrinsic aqueous solubility of GFB limits its direct use. Therefore, Cremophor RH 40 improves GFB solubility in water by forming a micellar microenvironment, enhancing fluorescence intensity while reducing organic-solvent consumption [32].

Thus, the improved sensitivity of the proposed method is attributed to the combined effects of dielectric stabilization, micellar solubilization, and reduced non-radiative deactivation in the aqueous Cremophor RH 40 medium.

### 2.2. Fluorescence Characteristics

#### 2.2.1. Effect of Surfactant Systems

**Figure 5:**
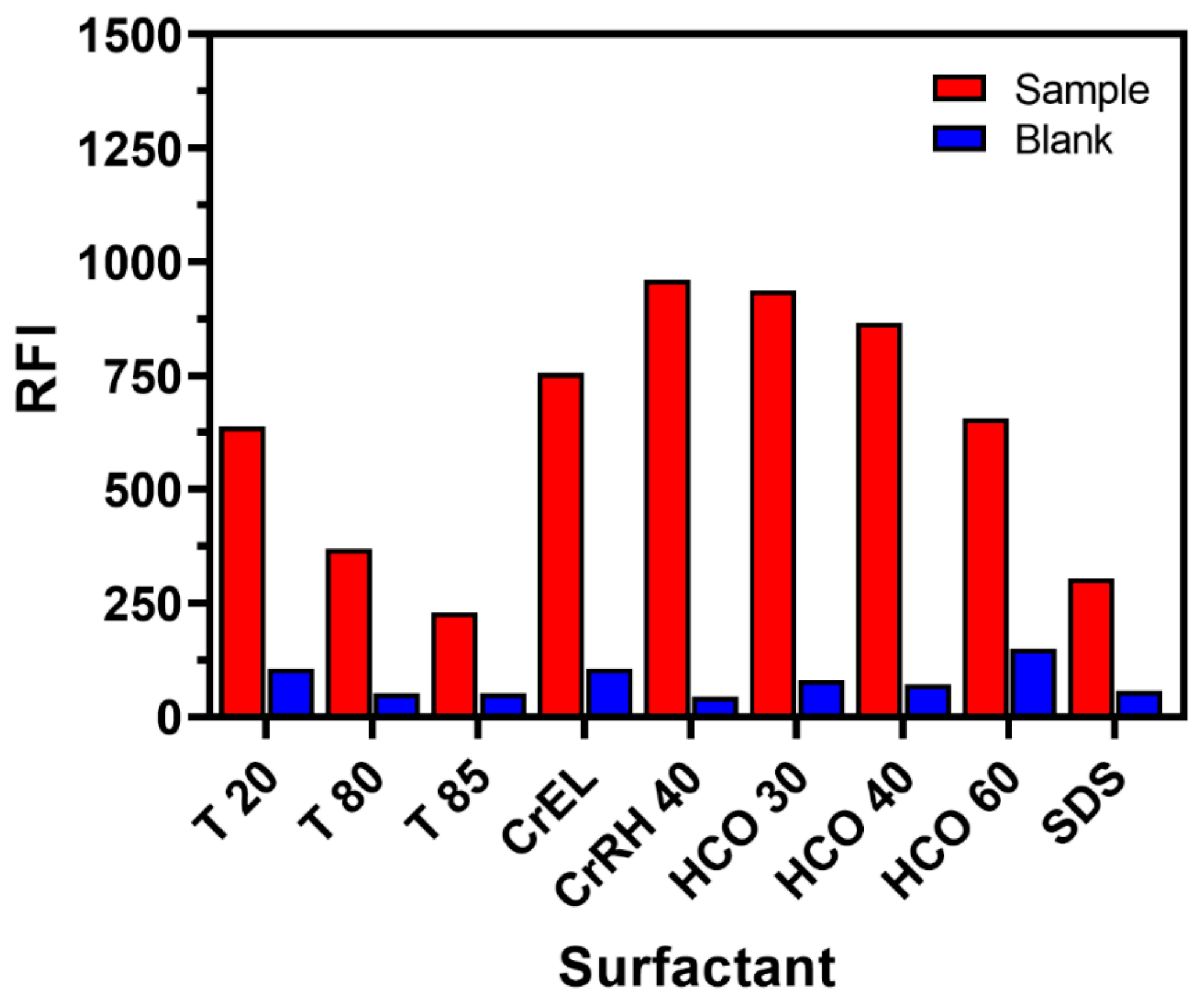
Effect of different organized media (each at a concentration listed in Table 3) on the RFI of GFB (500 ng/mL). Red columns: GFB + surfactant; blue columns: surfactant blank.

Nine surfactant systems (non-ionic and anionic surfactants) were screened at concentrations slightly above their respective critical micelle concentrations (CMC, Table 3). As shown in Figure 5, all surfactants enhanced the RFI of GFB relative to the surfactant-free blank, confirming that micellization is the general mechanism of fluorescence enhancement [33]; however, the magnitude of enhancement varied considerably with head-group chemistry and micelle polarity. Cremophor RH 40 gave the highest RFI, followed closely by HCO-30 and Cremophor EL, while the ionic surfactant SDS and the Tween surfactants with different aggregation behavior (80, 85) gave comparatively modest enhancement. This trend is consistent with the non-ionic polyoxyethylated castor-oil surfactants forming larger, more hydrophobic micellar cores capable of more effectively sequestering the moderately lipophilic GFB molecule away from the aqueous phase, whereas the anionic head group of SDS may additionally quench fluorescence through electrostatic or heavy-atom-independent non-radiative pathways. Cremophor RH 40 was therefore selected as the organized medium for all subsequent work [33].

#### 2.2.2. Effect of Surfactant (Cremophor RH 40) Concentration

**Figure 6:**
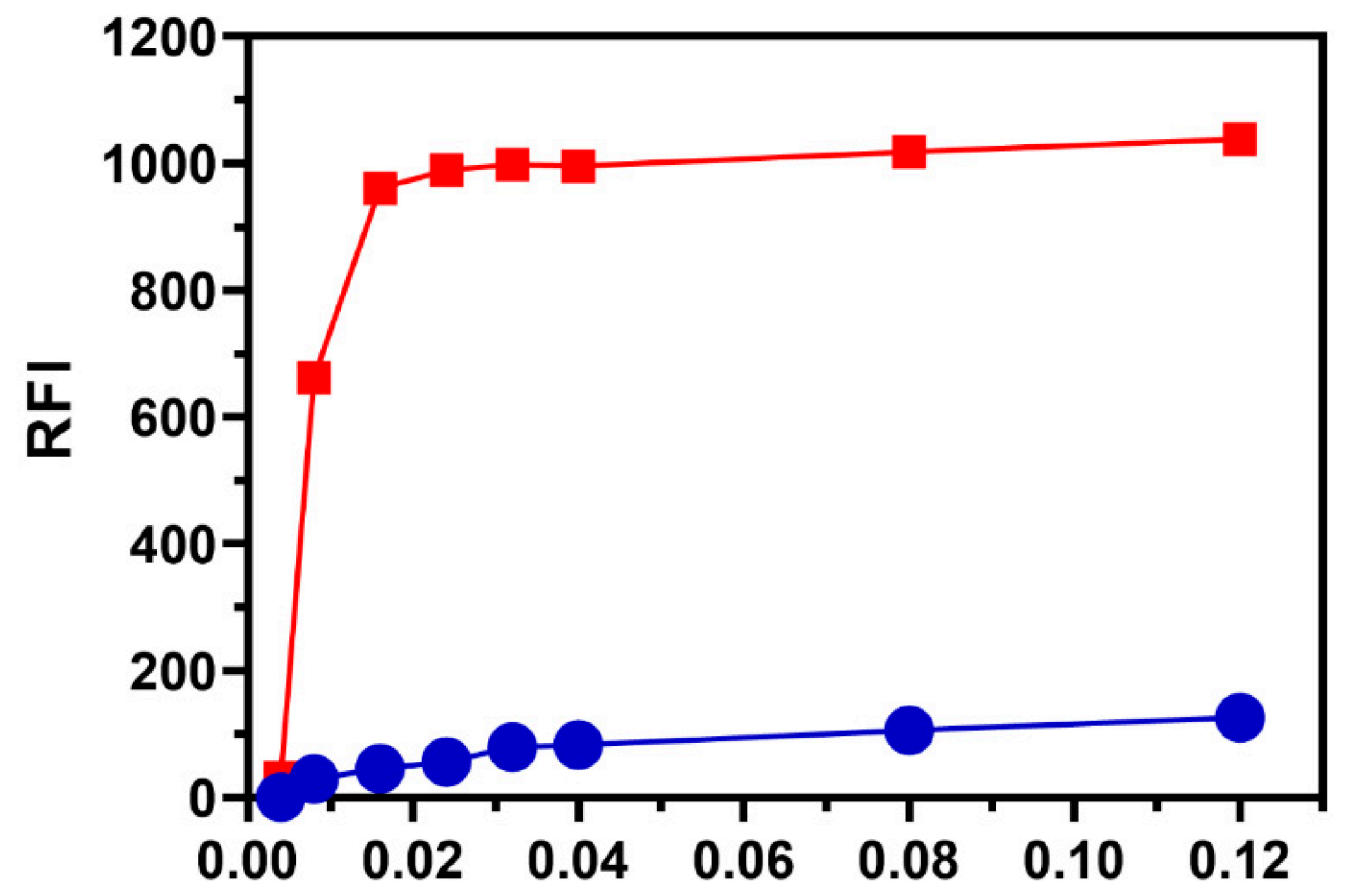
Effect of Cremophor RH 40 concentration (0.004–0.12 mM) on the fluorescence intensity of GFB (500 ng/mL). Red: GFB + surfactant; blue: surfactant blank.

Holding GFB concentration constant at 500 ng/mL, the Cremophor RH 40 concentration was varied from 0.004 to 0.12 mM, spanning the surfactant’s CMC (≈0.016 mM). RFI rose sharply as the surfactant concentration approached and exceeded the CMC, then plateaued from about 0.02 mM onward (Figure 6), consistent with the onset of micelle formation: below the CMC, surfactant monomers cannot form the hydrophobic domains needed to sequester GFB, whereas above it, additional surfactant simply increases the number of micelles without further improving partitioning of the drug already partitioned into available micelles. A concentration of 0.02 mM Cremophor RH 40 was selected as optimal, providing maximal RFI while minimizing reagent consumption and background fluorescence from the blank, which itself rose only gradually across the concentration range studied.

#### 2.2.3. Effect of pH

**Figure 7:**
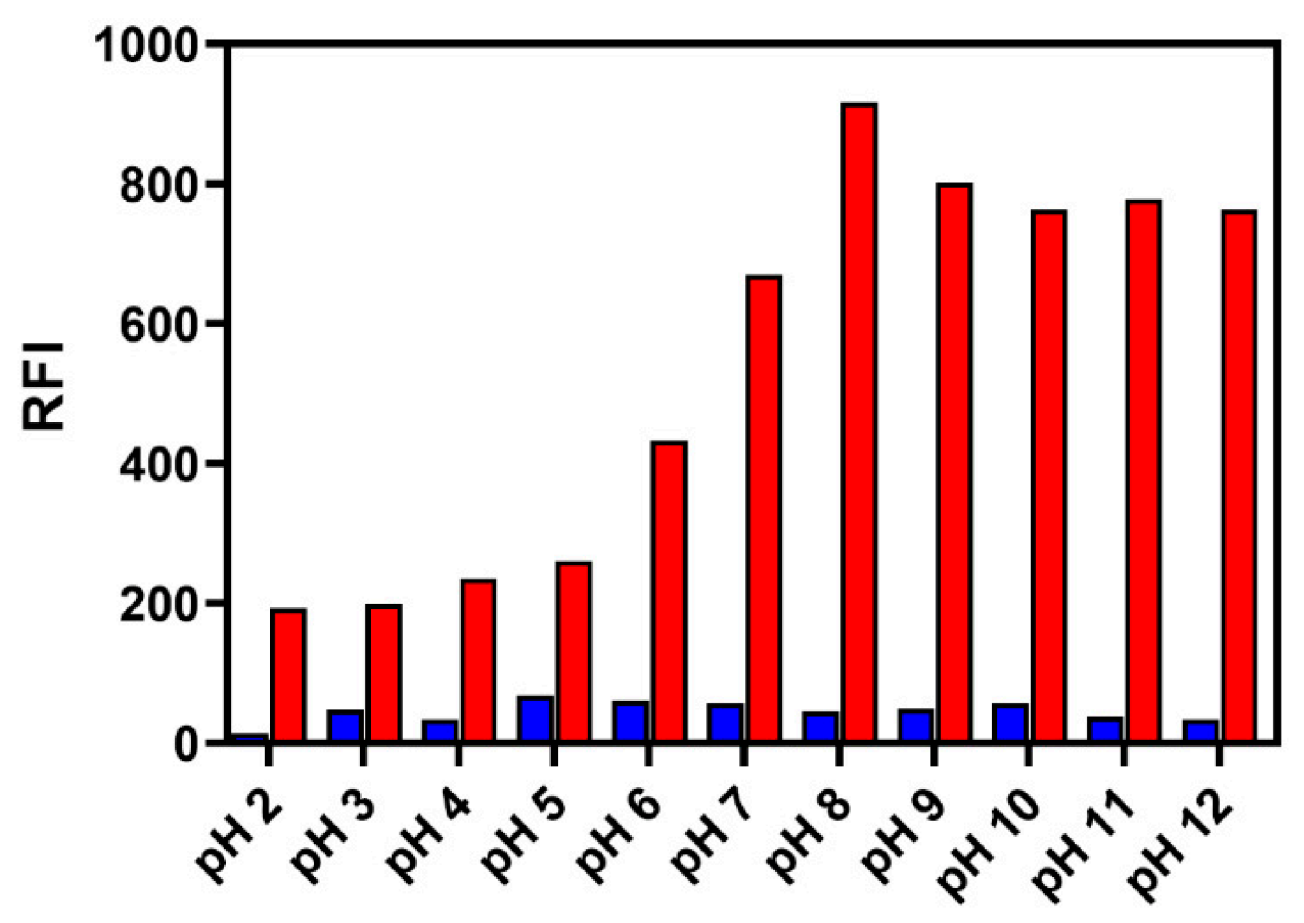
Effect of pH (2–12) on the RFI of 500 ng/mL GFB in 0.005% *w*/*v* Cremophor RH 40 (buffer type/pH varied as phosphate, citrate, and borate solutions). Red: GFB + surfactant; blue: surfactant blank.

The effect of pH (2–12) on the RFI of GFB, GFB–Cremophor RH 40, and Cremophor RH 40 alone was examined using phosphate, citrate, and borate buffers spanning the required ranges (Figure 7). The RFI of the GFB–surfactant system increased steadily with increasing pH, was essentially highest near pH 8 and lower at more acidic or alkaline values, and fell markedly below pH 6 (most noticeably at pH 4). This behavior is consistent with the ionization state of the morpholine side-chain of GFB: under more basic conditions the amine is progressively deprotonated, increasing the hydrophobicity of the molecule and favoring its partitioning into the Cremophor RH 40 micelle, whereas at low pH the protonated, more hydrophilic species partitions less efficiently into the micellar phase. The RFI of the surfactant blank remained essentially unchanged across the whole pH range, which is analytically advantageous because it maintains a relatively stable blank response, essentially pH-independent over the working range [34].

#### 2.2.4. Effect of Buffer Volume

**Figure 8:**
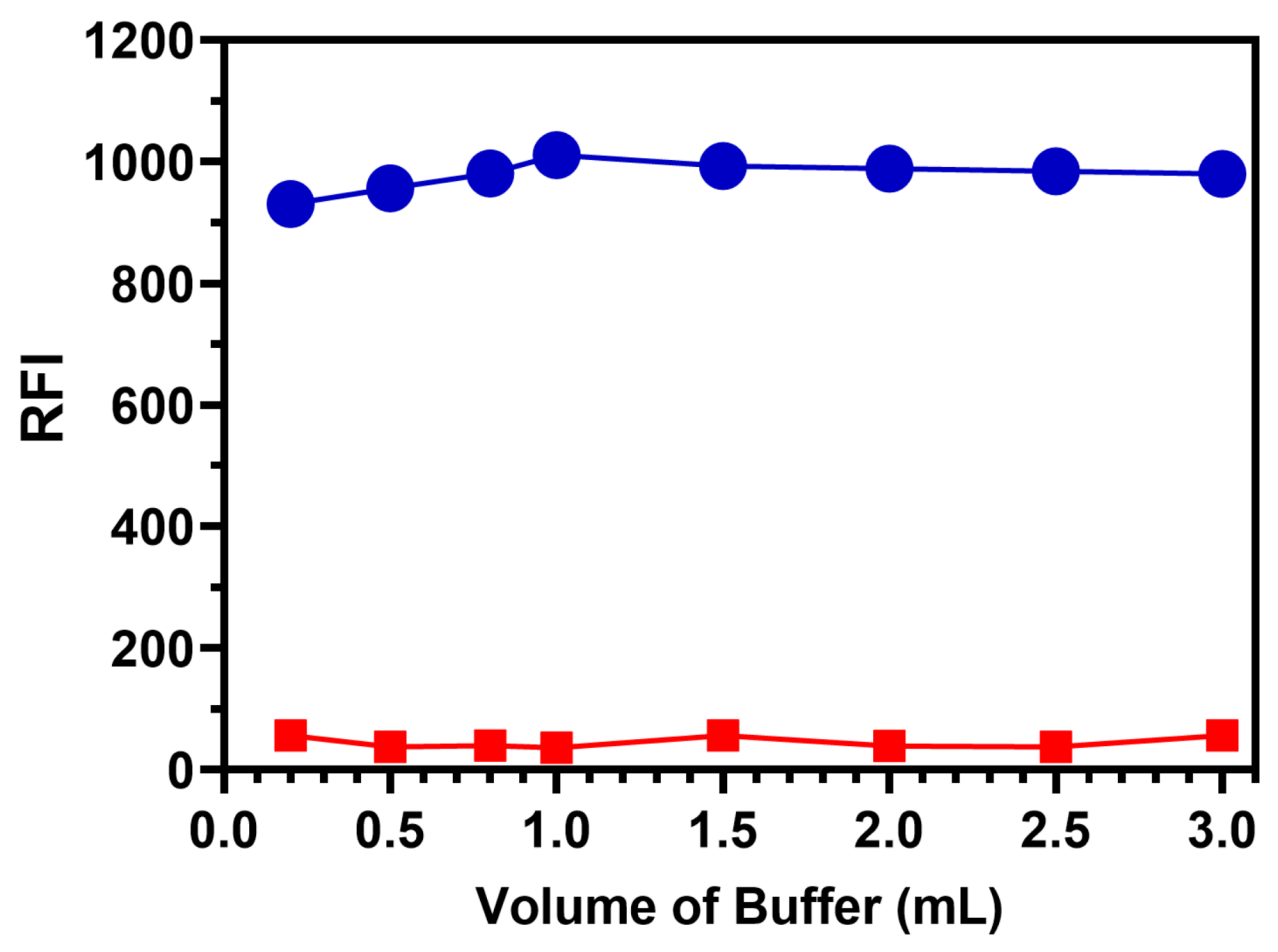
Effect of the volume of 0.1 M borate buffer (pH 8.0) on the RFI of GFB (500 ng/mL) in the presence of Cremophor RH 40. Blue: GFB–Cremophor RH 40; Red: surfactant blank.

The volume of buffer added was varied to establish the minimum amount required for maximal, stable RFI; RFI increased up to a threshold buffer volume and then remained essentially constant on further addition, indicating that the system was no longer buffer-limited beyond this point. On this basis, 1 mL of buffer was adopted for all subsequent experiments (Section 2.4). The corresponding RFI–buffer volume profile is shown in Figure 8. Based on these results, 1.0 mL of 0.1 M borate buffer at pH 8.0 was selected for all subsequent experiments (Section 3.3.3).

#### 2.2.5. Effect of Diluting Solvent

**Figure 9:**
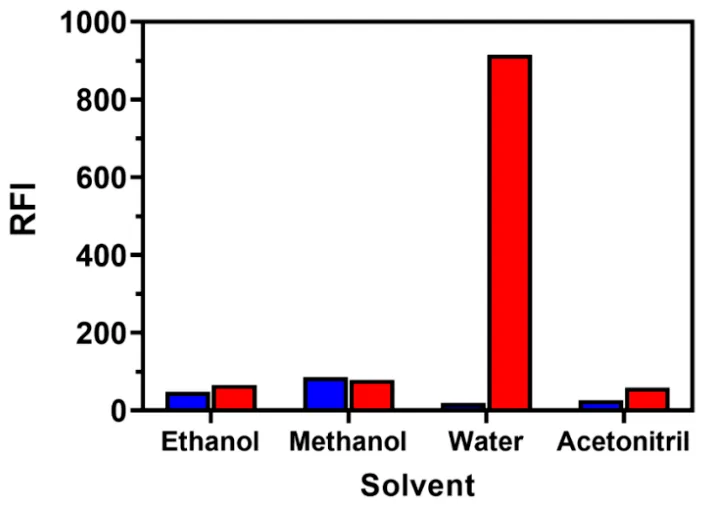
Effect of diluting solvent on the RFI of GFB (500 ng/mL). Red: GFB–Cremophor RH 40; blue: GFB alone.

Water, ethanol, methanol, and acetonitrile were compared as diluting solvents for the final measurement step (Figure 9). Water gave the highest RFI response under the tested conditions for the GFB–Cremophor RH 40 system, substantially outperforming all three organic solvents. This is consistent with the mechanism proposed above: dilution with a water-miscible organic solvent partially disrupts the Cremophor RH 40 micelles or alters their polarity, reducing the efficiency with which GFB is shielded from quenching interactions, whereas dilution with water preserves the intact micellar microenvironment. Because water also eliminates the need for any organic solvent in the final assay, it was adopted as the diluting solvent, reinforcing the green profile of the method (Section 2.6).

#### 2.2.6. Response-Surface (Box–Behnken) Confirmation of the Univariate Optimum

**Figure 10:**
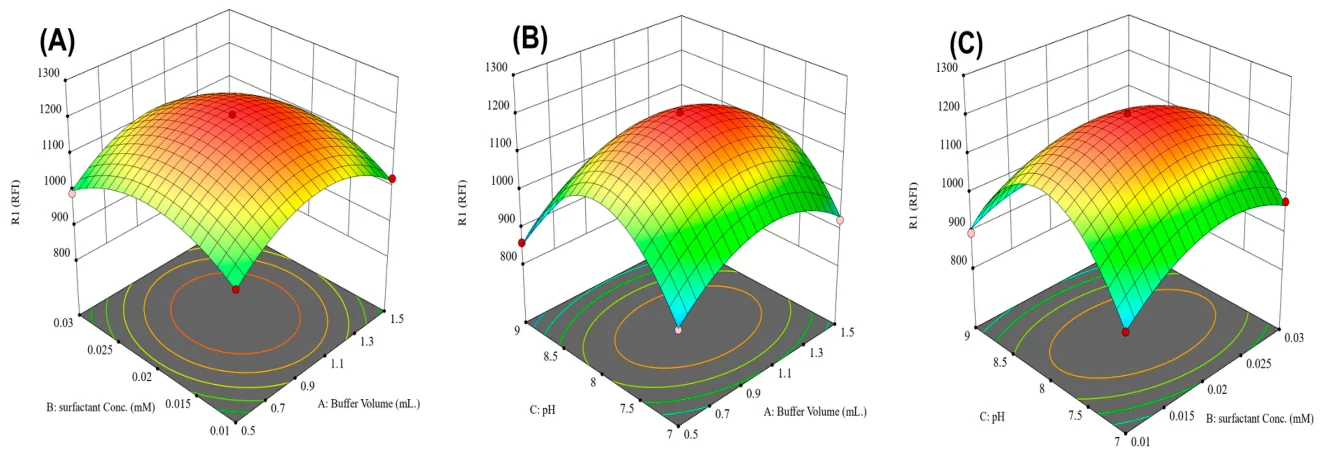
Response-surface plots showing the combined effects of buffer volume, Cremophor RH 40 concentration, and buffer pH on the relative fluorescence intensity (RFI) of GFB: (**A**) buffer volume and Cremophor RH 40 concentration at constant pH; (**B**) buffer volume and pH at constant Cremophor RH 40 concentration; and (**C**) Cremophor RH 40 concentration and pH at constant buffer volume. The pink and red dots represent the experimental design points used to generate the response surfaces.

Following the preliminary univariate experiments, buffer volume (A), Cremophor RH 40 concentration (B), and buffer pH (C) were simultaneously evaluated using a three-factor response-surface design, with relative fluorescence intensity (RFI) as the measured response. The design comprised 13 runs (a three-factor Box–Behnken arrangement with a single centre point), and the fitted surfaces are shown in Figure 10. The resulting full quadratic model was statistically significant (F = 83.26, *p* = 0.0019) and exhibited a strong descriptive fit, with R^2^ = 0.9960, adjusted R^2^ = 0.9840, a coefficient of variation of 1.38%, and an adequate precision value of 33.4298 (Table 4) [35].

The fitted quadratic equation in terms of coded variables was as follows:

$$\begin{array}{cc} \mathrm{RFI}= & 1205.00+8.00A-0.375B-33.62C\\ & -3.00\mathrm{AB}-16.50\mathrm{AC}-41.75\mathrm{BC}\\ & -110.12A^{2}-88.87B^{2}-218.88C^{2} \end{array}$$

ANOVA showed that the linear effect of pH (C) was statistically significant (*p* = 0.0054), whereas the linear effects of buffer volume (A) and Cremophor RH 40 concentration (B) were not significant (*p* > 0.05). A significant interaction was observed between Cremophor RH 40 concentration and pH (BC, *p* = 0.0078), indicating that the effect of Cremophor RH 40 concentration on RFI depended on the pH of the analytical medium. In contrast, the AB and AC interactions were not statistically significant.

All three quadratic terms were statistically significant, with *p*-values of 0.0010, 0.0020, and 0.0001 for A^2^, B^2^, and C^2^, respectively. Their negative coefficients demonstrated downward curvature in the response surfaces, indicating that intermediate factor levels produced higher fluorescence responses than the investigated extreme levels. The magnitude of the quadratic coefficients followed the order ∣C^2^∣ > ∣A^2^∣ > ∣B^2^∣, showing that pH exerted the strongest nonlinear influence on RFI. This behavior may reflect pH-dependent changes in gefitinib ionization, micellar partitioning, and nonradiative relaxation (Table 4).

The significant BC interaction further demonstrated that Cremophor RH 40 concentration and pH should be controlled jointly rather than optimized independently. The response-surface findings therefore complemented the preliminary univariate experiments by identifying significant curvature and interaction effects and supporting the selection of operating conditions located near the central high-response region.

Despite the strong descriptive fit of the model, the predicted R^2^ and PRESS statistics could not be calculated because at least one experimental point had a leverage value of 1.000. In addition, a formal lack-of-fit test was unavailable, and the model contained only three residual degrees of freedom. Nevertheless, three independent confirmation experiments conducted under the selected conditions showed close agreement between the predicted and observed RFI values, with individual prediction errors of 0.954%, 0.914%, and 0.515% and a mean absolute prediction error of 0.794% (Table 5). These results support the local adequacy of the model at the selected operating point but do not establish its predictive performance throughout the entire experimental domain. Accordingly, the model was used to support the identification of a high-response region and the selection of the analytical conditions, rather than as evidence of complete predictive validation. The investigated experimental region was used exclusively for analytical procedure optimization and should not be interpreted as a formally established analytical procedure design region or as providing regulatory flexibility around the selected conditions. It should be emphasized that the derived regression equation represents a local model applicable only within the immediate vicinity of the optimized operating conditions (buffer volume 1.0 mL, Cremophor RH 40 concentration 0.005% *w*/*v*, and pH 8.0), where its predictive performance was experimentally verified. Accordingly, the model should not be used to predict relative fluorescence intensity (RFI) at other combinations of the investigated factors, to interpolate across the entire experimental domain, to extrapolate beyond the studied factor ranges, or to establish a design space or method operable design region (MODR). The equation is therefore intended solely as a descriptive tool for characterizing the local response behavior around the selected operating point. Because no lack-of-fit test, predicted R^2^ or PRESS statistic could be computed, the only evidence of model adequacy available is the agreement of the three confirmation runs at that single point, and the model’s coefficients should be read as indicating the relative importance and the sign of the effects rather than as a predictive calibration of the response surface. A design with additional centre points and an augmented set of runs would be required before any predictive or regulatory use of the model could be claimed.

$$\mathrm{Prediction error}(\%)=\frac{\left|\mathrm{Observed RFI}-\mathrm{Predicted RFI}\right|}{\mathrm{Predicted RFI}}$$

### 2.3. Method Validation

#### 2.3.1. Linearity and Sensitivity

**Figure 11:**
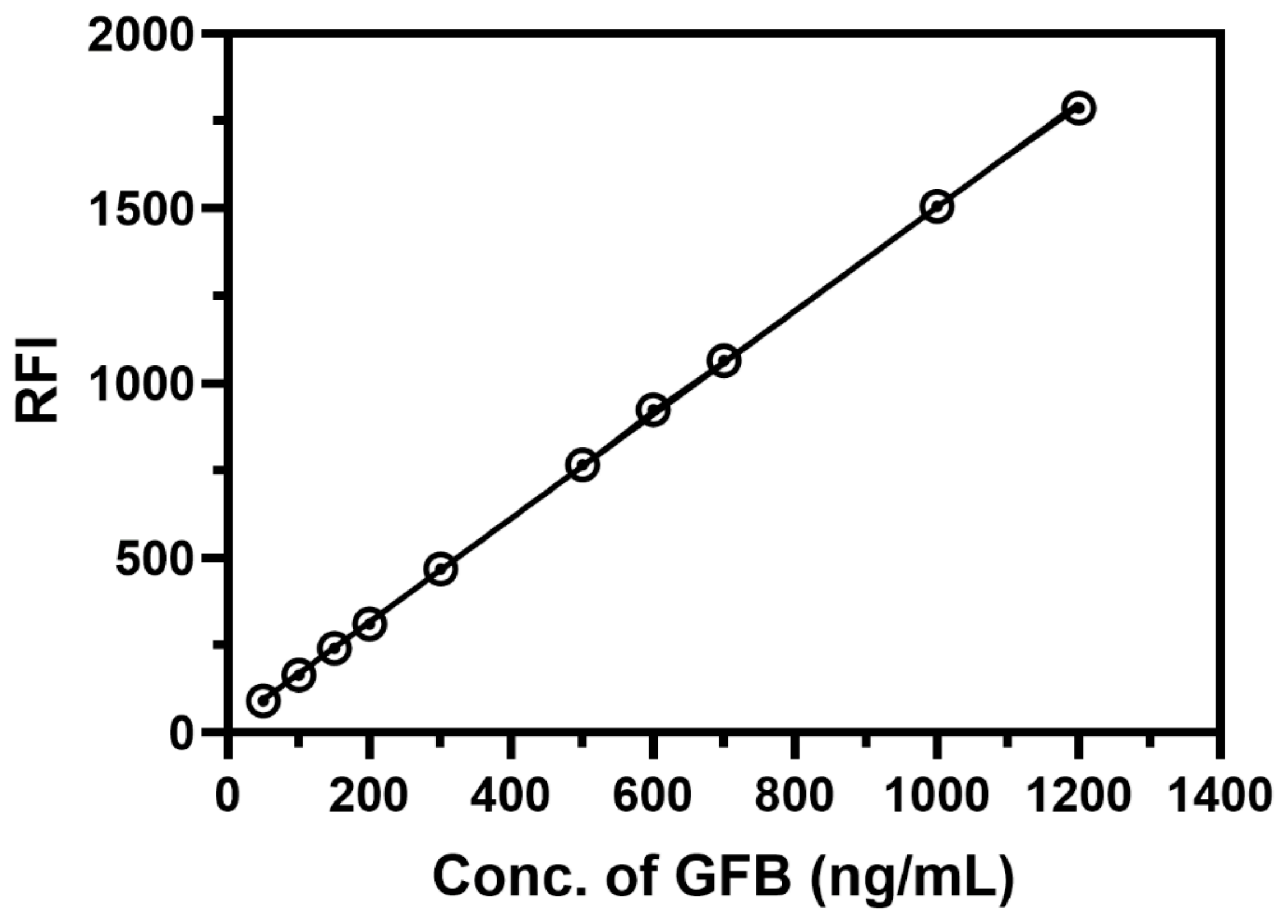
Calibration curve of GFB using the proposed micelle-enhanced spectrofluorimetric method, showing the linear relationship between RFI and GFB concentration over 50–1200 ng mL^−1^.

The linearity and sensitivity of the proposed spectrofluorimetric method for the determination of GFB were evaluated under the optimized experimental conditions described in Section 2.4. Fluorescence intensity was measured at an emission wavelength of 394 nm following excitation at 333 nm. The relative fluorescence intensity (RFI) values were plotted against the corresponding GFB concentrations. As summarized in Table 6, the method demonstrated a linear response over the concentration range of 50–1200 ng mL^−1^ using six calibration points (Figure 11).

Linear regression analysis of the calibration data produced the following equation:

RFI = 20.99 + 1.483*C*

where *C* is the concentration of GFB in ng mL^−1^, 20.99 is the intercept, and 1.483 is the slope of the calibration curve. The high correlation coefficient (*r* = 0.9997) confirmed the excellent linear relationship between fluorescence intensity and GFB concentration within the investigated range.

The standard deviation of the residuals (*S_y_*_/*x*_) was 10.43, while the standard deviations of the intercept (*S_a_*) and slope (*S_b_*) were ±3.098 and ±0.0051, respectively. These relatively low values indicate limited dispersion of the experimental data around the regression line and satisfactory precision of the estimated regression parameters. Furthermore, the low values of %RSD and % error, which were 0.34% and 0.343%, respectively, confirmed the reliability and consistency of the proposed method.

The sensitivity of the method was further evaluated by determining the limit of detection (LOD) and limit of quantitation (LOQ). The LOD and LOQ were found to be 6.89 and 20.89 ng mL^−1^, respectively, demonstrating that the proposed spectrofluorimetric method provides adequate sensitivity for the detection and quantitative determination of GFB at low concentration levels [36].

The precision associated with the calibration measurements was satisfactory, as indicated by a %RSD of 0.34%, while the corresponding relative error was 0.343%, demonstrating low variability and minimal deviation between the experimental and nominal values under the optimized analytical conditions.

#### 2.3.2. Sensitivity

The LOD and LOQ were calculated according to ICH Q2(R2) [37,38]:

$$\mathrm{LOD}=3.3\times \frac{\sigma }{S};$$

$$\mathrm{LOQ}=10\times \frac{\sigma }{S}$$

where σ is the standard deviation of the intercept and S is the slope of the calibration line (Table 6). The resulting LOD (6.89 ng/mL) and LOQ (20.89 ng/mL) confirm that the method is sufficiently sensitive for tablet-level GFB assay and tablet assay work, where label-claim concentrations are typically one to two orders of magnitude above the LOQ.

Although the calculated LOQ (20.89 ng mL^−1^) lies below the lowest calibration level, 50 ng mL^−1^ was deliberately retained as the lower limit of the validated range for three reasons. First, the σ/S expressions of ICH Q2(R2) yield an estimate derived from the variance of the regression intercept; that estimate is an extrapolation below the range actually studied and, under ICH Q2(R2), has to be confirmed by the analysis of samples at or near the estimated limit before it can be reported as a validated concentration. No accuracy or precision data were generated at 20.89 ng mL^−1^, so this figure is reported here as a calculated sensitivity descriptor only and not as a reportable limit. Second, 50 ng mL^−1^ is about 2.4 times the calculated LOQ and about 7 times the LOD, which provides the signal margin needed to absorb the day-to-day variability of the buffer and blank response that was observed in the intermediate-precision study (Section 2.3.3); a reportable limit set at the calculated LOQ would have no such margin. Third, the concentration interval required for the intended purpose of the procedure—the assay of 250 mg gefitinib tablets after dilution—is 100–500 ng mL^−1^, so the validated range already extends a factor of two below the lowest routine working concentration and there is no analytical need to claim a lower limit. Extending the validated range below 50 ng mL^−1^ would require additional accuracy and precision experiments at that level, which were not part of the present work.

#### 2.3.3. Accuracy and Precision

The accuracy and precision of the proposed spectrofluorimetric method for the determination of GFB were evaluated by analyzing GFB bulk powder samples at three concentration levels representing the low, medium, and high ranges of the calibration curve. The selected concentrations were 200, 500, and 1000 ng mL^−1^. At each concentration level, six independent sample solutions were prepared separately from the working standard solution. Each solution underwent the complete analytical procedure, including separate aliquoting, addition of Cremophor RH 40 and buffer, dilution to volume, mixing, equilibration, and fluorescence measurement. Thus, *n* = 6 represents six independent preparations rather than six replicate instrumental readings of a single prepared solution. Intraday accuracy and precision were assessed by analyzing these concentrations within the same day, whereas interday accuracy and precision were evaluated over three consecutive days. Accuracy was expressed as percentage found, calculated by comparing the measured concentration with the nominal concentration, while precision was expressed as the percentage relative standard deviation (%RSD).

As shown in Table 7, the intraday percentage found values ranged from 97.55% to 101.32%, with %RSD values between 0.33% and 1.52%. For the interday study, the percentage found values ranged from 99.18% to 102.22%, with %RSD values ranging from 1.61% to 3.24%. The corresponding percentage error values were low, ranging from 0.52% to 2.45% for intraday analysis and from 0.82% to 2.22% for interday analysis. With the single exception discussed below, these results support the accuracy and repeatability of the proposed method for the quantification of GFB in bulk form.

The one result that did not meet the predefined precision criterion was the inter-day %RSD of 3.24% at 500 ng mL^−1^, and it is retained and discussed here rather than excluded. Three observations bear on its interpretation. First, the deviation is not concentration-dependent: the inter-day %RSD values were 1.61% at 200 ng mL^−1^ and 1.80% at 1000 ng mL^−1^, so a loss of precision confined to the middle of the range cannot be attributed to a systematic sensitivity, curvature or blank-subtraction effect, and the accuracy at 500 ng mL^−1^ (99.18% found, 0.82% error) was in fact the best of the three levels. Second, an %RSD estimated from six independent preparations is itself an imprecise statistic: the 95% confidence interval of the underlying population %RSD, obtained from the χ^2^ distribution with five degrees of freedom, is approximately 2.0–7.9%. The observed value therefore shows that intermediate precision at this level has not been demonstrated with confidence, rather than that it is demonstrably unacceptable, and it would be incorrect to draw either a favourable or an unfavourable firm conclusion from a single estimate with so wide an interval. Third, the most plausible physical sources are the factors that both the response-surface analysis and the robustness study identified as critical. Buffer pH had by far the largest quadratic coefficient in the response-surface model, and a deviation of only ±0.3 pH unit changed the recovery by up to about 5 percentage points (Table 8); since the borate buffer was prepared freshly on each of the three days, small differences in its adjusted pH are the single most likely contributor to the between-day dispersion. A second candidate is ageing of the aqueous Cremophor RH 40 stock solution, in which slow hydrolysis of the fatty-acid esters and progressive change in the aggregation state can alter the micellar microenvironment and therefore the enhancement factor, and a third is the volumetric transfer of the small aliquot of organic working standard, whose relative error is greatest at the intermediate dilution step. Accordingly, the following mitigations are proposed for routine laboratory use and are recommended as part of the analytical control strategy: prepare the borate buffer fresh and verify its pH to within ±0.05 unit immediately before use; prepare the Cremophor RH 40 solution fresh each working day, or establish and qualify a defined in-use period for it; keep the 5 min equilibration time constant with a timer; construct a fresh calibration curve on each day of analysis instead of relying on a stored curve; and analyse a mid-range system-suitability standard, with an acceptance window of ±3% of the expected RFI, before each sample set. Mid-range intermediate precision should be re-established under these controls, preferably in a formal design that also varies analyst and instrument, before the procedure is used for release testing; this limitation is stated in Section 2.7 and in the Conclusions.

#### 2.3.4. Robustness

Robustness was assessed by making small, deliberate variations to the optimized conditions—Cremophor RH 40 volume (±50 µL), pH (±0.3 units), and buffer volume (±50 µL)—and measuring the resulting recovery (Table 8). All variations produced recoveries within the range typically considered acceptable for a robust method (97–105%), indicating that small, unavoidable variability during routine use of the method is unlikely to compromise its performance. The pH variation was applied to the 0.1 M borate buffer over the interval 7.7–8.3, and the quoted pH values refer to that buffer, which was adjusted and verified with a calibrated pH meter before it was added to the assay flask (Section 3.3.3). The only variation that approached the acceptance boundary was pH 8.3, for which the mean recovery of 105.05% lies at or marginally above the upper limit; pH is therefore the only parameter of the three that has to be controlled tightly, and a tolerance of ±0.1 pH unit is recommended for routine use rather than the ±0.3 unit tested here.

$$\%\mathrm{RSD}=\frac{\mathrm{SD}}{\mathrm{mean recovery}}\times 100.$$

Each value represents 6 independently prepared analytical solutions. Each solution was processed separately through the complete analytical procedure, and repeated instrumental measurements of a single prepared solution were not considered independent replicates.

The predefined acceptance criteria were a mean recovery within 95.0–105.0% and a %RSD of not more than 3.0%.

#### 2.3.5. Specificity and Matrix Selectivity

The specificity of the proposed spectrofluorimetric method was evaluated by investigating the possible interference from common tablet excipients, including lactose, magnesium stearate, microcrystalline cellulose, and starch. A placebo mixture containing these excipients was prepared and subjected to the same sample-preparation and analytical procedure used for the tablet formulation. No significant fluorescence signal was observed from the placebo solution at λ_ex_/λ_em_ = 333/394 nm, indicating that the excipients did not interfere with the native fluorescence of GFB or with its micellar enhancement in Cremophor RH 40. This supports the selectivity of the proposed method for GFB determination in the intact tablet matrix investigated.

The excipients tested were selected as generic tablet diluents, disintegrants and lubricants, and their relationship to the actual formulation should be made explicit. The qualitative composition of the Iressa^®^ 250 mg film-coated tablet is lactose monohydrate (163.5 mg per tablet), microcrystalline cellulose, croscarmellose sodium, povidone K29-32, sodium laurilsulfate and magnesium stearate in the core, with hypromellose, macrogol 300, titanium dioxide (E171) and yellow and red iron oxides (E172) in the film coat (Section 3.2). None of these components contains a conjugated aromatic fluorophore that absorbs at 333 nm and emits near 394 nm, and the placebo blend covered the two components present in the largest amounts. One component nevertheless deserves specific comment: sodium laurilsulfate is itself a micelle-forming surfactant and was shown in Section 2.2.1 to enhance the fluorescence of GFB, so in principle a co-extracted portion of it could contribute to the signal. In practice it cannot, because of the extent of dilution involved. A tablet containing 250 mg of gefitinib is ultimately measured at 50–1200 ng mL^−1^, a dilution of more than five orders of magnitude, so even if the entire surfactant content of the tablet were co-extracted, the concentration of sodium laurilsulfate in the final measurement solution would remain several orders of magnitude below its critical micelle concentration of about 8 mM (Table 3), and no micellar contribution is possible. The absence of any systematic positive bias in the tablet recovery data (Table 9) is consistent with this expectation and provides the direct experimental evidence that the intact formulation matrix does not interfere. The titanium dioxide and iron oxide pigments of the film coat are insoluble in the extraction medium and are removed at the filtration step (Section 3.3.2).

The accuracy of the method was further confirmed by recovery studies using both pure GFB bulk powder and tablet samples. Known concentrations of GFB were analyzed at three levels: 100, 250, and 500 ng mL^−1^. As shown in Table 9, the mean recovery for bulk powder was 99.75 ± 1.19%, while the mean recovery for tablet samples was 100.21 ± 1.86%. These results demonstrate that the proposed method provides accurate recovery in both pure drug and pharmaceutical dosage-form matrices. The close agreement between the taken or added and the found concentrations indicates that the excipients of the investigated product do not significantly affect the analytical response of GFB.

Two limitations of this selectivity assessment must, however, be stated explicitly, since neither is addressed by the excipient and recovery experiments described above. First, the procedure is non-separative: any co-extracted species that absorbs near 333 nm and emits near 394 nm contributes additively to the measured RFI, and the method has no means of distinguishing such a contribution from gefitinib itself. This applies in particular to process-related impurities and to degradation products that retain the 4-anilinoquinazoline fluorophore. The published forced-degradation study of gefitinib shows that the principal degradation products arise from modification of the morpholinopropoxy side chain and of the halogenated anilino substituent while the quinazoline ring system is preserved [11]; their native fluorescence is therefore expected to overlap that of the parent drug, and the method is selective for the intact tablet matrix but is not specific with respect to degradants and is not stability-indicating (Section 2.7). Second, only the single-entity Iressa^®^ product was examined. Gefitinib is approved and administered as monotherapy and is not co-formulated, so co-administered drugs are not present in the dosage unit being assayed and cannot interfere with the tablet assay as performed here. They could, however, be present in samples of biological origin, in compounded or co-packaged products, or in counterfeit material, and trace quinazoline-related synthesis impurities in lower-grade generic products would not be resolved either. Any extension of the procedure to plasma, urine, fixed-dose combinations or unknown provenance material would therefore require selectivity to be re-established in the new matrix and would most probably require a resolution-enhancing fluorimetric mode, such as synchronous or first-derivative synchronous fluorescence, or a separation step, rather than the single wavelength-pair measurement used here.

### 2.4. Assay of Gefitinib in Commercial Tablets

The applicability of the proposed micelle-enhanced spectrofluorimetric method was evaluated by determining gefitinib in commercially available Iressa^®^ tablets labeled to contain 250 mg of gefitinib per tablet. Ten tablets were collectively weighed, finely powdered, and mixed to obtain a homogeneous composite sample. An accurately weighed portion of the pooled tablet powder equivalent to 100 mg of gefitinib was extracted and diluted according to the procedure described in Section 3.3.2. The resulting sample solutions were further diluted to concentrations within the validated analytical range of 50–1200 ng mL^−1^ before fluorescence measurement.

The proposed method produced a mean gefitinib assay of 100.21 ± 1.86% of the label claim, corresponding to 250.53 ± 4.65 mg per tablet. The result was close to the declared content of 250 mg per tablet and supports the applicability of the method to the assay of gefitinib in the investigated commercial tablet product. Because the analysis was performed using a pooled powder prepared from ten tablets, the result represents an average tablet assay and should not be interpreted as an assessment of tablet assay among individual dosage units.

The observed recovery of the tablet sample was also close to 100%, indicating that the common tablet excipients did not significantly affect the fluorescence response of GFB in the Cremophor RH 40 micellar medium.

The tablet-assay results were descriptively compared with those reported for the previously published spectrofluorimetric method for gefitinib [7]. The proposed method produced a mean assay of 100.21 ± 1.86% of the label claim, whereas the reported method gave 100.47 ± 0.42%. Because these results were obtained in different studies using different tablet products, laboratories, instruments, and replicate designs, the comparison is descriptive only. No statistical equivalence or superiority is claimed.

### 2.5. Comparison with the Reported Spectrofluorimetric Method for Gefitinib

The proposed method was compared with the previously reported spectrofluorimetric method for gefitinib determination [7]. Both methods measure the native fluorescence of gefitinib without derivatization. However, the reported method uses ethanol as the measurement medium, whereas the proposed method uses an aqueous borate-buffered medium containing Cremophor RH 40.

The proposed method showed a wider and more sensitive working range of 50–1200 ng mL^−1^, compared with 1000–3500 ng mL^−1^ for the reported method. It also provided lower LOD and LOQ values of 6.89 and 20.89 ng mL^−1^, respectively, compared with 29.9 and 90.6 ng mL^−1^ for the reported method. This improvement is attributed to the micellar enhancement of gefitinib fluorescence by Cremophor RH 40.

The comparison of the two fluorescence methods is therefore presented first. The key parameters of the two fluorimetric procedures are collected in Table 10.

The two procedures differ principally in the medium in which the final measurement is made. The reported method measures the fluorescence of gefitinib in ethanol, that is, in a homogeneous organic solvent, whereas the present method measures it in an aqueous 0.1 M borate buffer (pH 8.0) containing 0.005% *w*/*v* Cremophor RH 40, in which the drug is incorporated into a non-ionic micellar pseudophase. Three consequences of this difference are visible in Table 10. First, the analytical wavelengths are not transferable between the two procedures: incorporation into the micelle red-shifts the excitation maximum by more than 40 nm relative to bulk solvents, which supports the selection of 333 nm for excitation and 394 nm for emission measurement in the present method (Section 2.1.1). Second, the micellar medium shields the excited fluorophore from aqueous and hydrogen-bonding quenching and restricts non-radiative relaxation, which is the physical basis of the lower limits of detection and quantification reported here. Third, the measurement itself is performed in an essentially aqueous medium, which is the principal source of the greenness advantage discussed in Section 2.6.

With respect to selectivity, the comparison is deliberately conservative. Neither method separates the analyte from co-extracted species, so neither can be expected to discriminate gefitinib from degradation products or from impurities that retain the 4-anilinoquinazoline fluorophore (Section 2.3.5). The present method does differ from the reported method in one respect that is relevant to matrix tolerance: because micellar enhancement acts selectively on species that partition into the hydrophobic micellar core, the response of the analyte is amplified while that of polar, non-partitioning matrix components is not, and the surfactant blank was shown to be essentially independent of pH across the range 2–12 (Section 2.2.3), so the analyte-to-background ratio and the stability of the blank are both improved relative to measurement in bulk ethanol. That is, however, an enhancement of sensitivity and of blank stability, not a separation, and it is not presented here as an improvement in specificity towards degradants. On the evidence available, the present method offers improved sensitivity and a greener measurement step, comparable accuracy in the intact tablet matrix, and no demonstrated improvement in selectivity towards degradation products.

### 2.6. Green Profile of the Method

**Figure 12:**
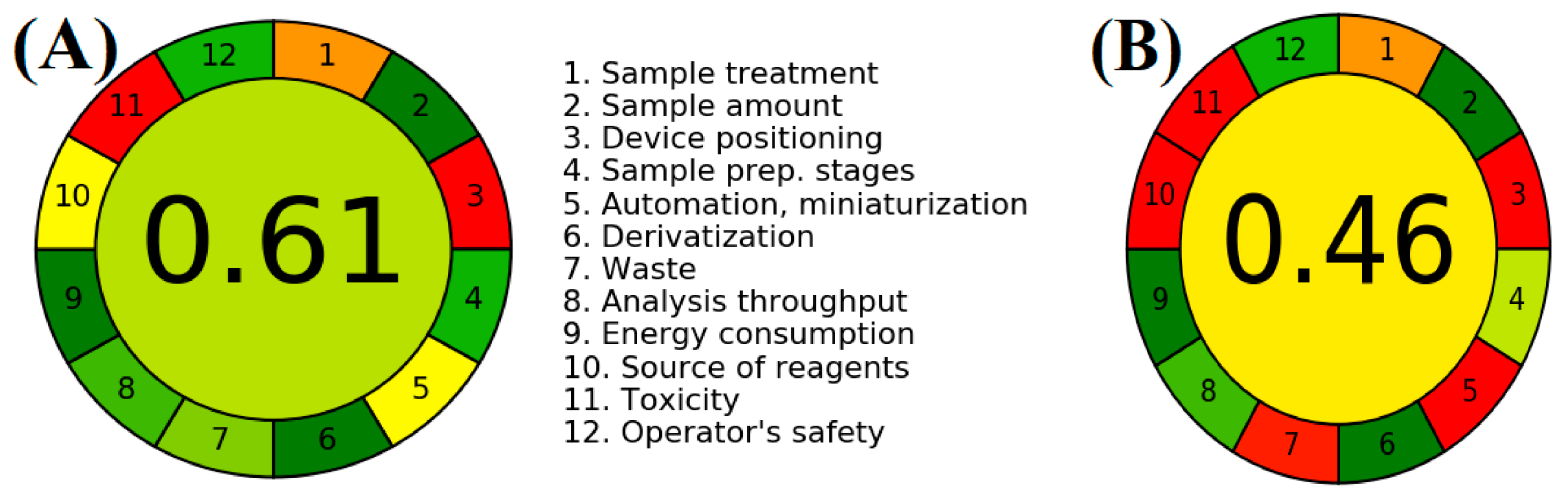
AGREE pictograms for (**A**) the proposed micelle-enhanced spectrofluorimetric method and (**B**) the reference spectrofluorimetric method, with overall scores of 0.61 and 0.46, respectively.

The AGREE assessment produced an overall score of 0.61 for the proposed method and 0.46 for the reference spectrofluorimetric method (Figure 12) [7]. In the pictogram for the proposed method, the red segments corresponding to principles 3 and 11 reflect offline analysis and the use of potentially hazardous organic solvents during sample preparation, respectively. By comparison, the reference method shows red segments for principles 3, 5, 7, 10, and 11, corresponding to offline analysis, limited automation and miniaturization, greater waste generation, limited use of renewable reagents, and reagent toxicity, respectively. Thus, the proposed procedure has a more favorable overall AGREE profile, primarily because of its aqueous micellar measurement medium and lower waste generation. Nevertheless, acetonitrile and DMSO are still required during sample preparation, and their replacement with safer solvents would further improve the environmental profile of the complete analytical procedure.

### 2.7. Limitations and Future Perspectives

The proposed method is intended for the average-content assay of intact gefitinib tablets and is not stability-indicating because it does not separate gefitinib from potentially fluorescent degradation products. The response-surface model was verified only locally at the selected conditions and should not be interpreted as defining a design space. In addition, intermediate precision at 500 ng mL^−1^ requires further evaluation, and content uniformity was not investigated because a pooled tablet sample was analyzed. The computational findings are interpretative rather than predictive, and the comparison with the reported spectrofluorimetric method [7] is literature-based. Future work will focus on matched-sample method comparison, improved intermediate precision, greener sample preparation, and application to content-uniformity and dissolution testing.

## 3. Materials and Methods

### 3.1. Apparatus

Fluorescence measurements were performed using a Jasco FP-8200 spectrofluorometer (Jasco Corporation, Hachioji-shi, Japan) equipped with a 150 W xenon lamp and 1-cm quartz cuvettes. Both excitation and emission slit widths were set at 5.0 nm. The instrument was routinely calibrated and its linearity was verified using a standard quinine sulfate solution. Fluorescence spectra were acquired and exported in ASCII format using Spectra Manager™ Suite (Jasco Corporation, Hachioji-shi, Japan). A Hanna pH meter (Cluj Napoca, Romania) was used for pH adjustment, while dissolution studies were performed using an eight-cup automated dissolution apparatus (Abbott Corporation, Franklin Road, NJ, USA).

### 3.2. Reagents and Materials

All chemicals were of analytical-reagent grade, and all solvents conformed to HPLC standards. GFB reference standard (purity > 99.00%) was obtained from LC Laboratories (New Boston, MA, USA). Iressa^®^ 250 mg film-coated tablets, each labelled to contain 250 mg of gefitinib (marketing-authorization holder AstraZeneca AB, Södertälje, Sweden; manufactured by AstraZeneca UK Limited, Macclesfield, UK), were obtained from a community pharmacy in Riyadh, Saudi Arabia (batch number: PF105; expiry date: 31 December 2021). According to the manufacturer’s product information, the tablet core contains lactose monohydrate (163.5 mg per tablet), microcrystalline cellulose, croscarmellose sodium, povidone K29-32, sodium laurilsulfate and magnesium stearate, and the film coat contains hypromellose, macrogol 300, titanium dioxide (E171) and yellow and red iron oxides (E172). For the placebo interference study, a laboratory blend of lactose, microcrystalline cellulose, magnesium stearate and starch was prepared and carried through the complete procedure. Polyoxyl 40 hydrogenated and polyoxyl 35 castor oils (Cremophor^®^ RH 40 and Cremophor^®^ EL, respectively) were supplied by BASF (Ludwigshafen, Germany) and used as 0.05% *w*/*v* aqueous solutions. Sodium dodecyl sulfate (SDS, 95% purity, Winlab, Birmingham, UK) was prepared as a 0.05% *w*/*v* aqueous solution. Tween 20, Tween 80, and Tween 85 (Techno Pharmchem, Bahadurgarh, Haryana, India) were used as 1% *v*/*v* aqueous solutions. Methanol and ethanol were from Prolabo (Fontenay-sous-Bois, France); acetonitrile was from Sigma-Aldrich (Taufkirchen, Germany).

Buffer components (boric acid, citric acid, sodium hydroxide, phosphoric acid, potassium chloride, potassium dihydrogen phosphate, disodium hydrogen phosphate) were of spectroscopic grade. The following buffers were freshly prepared: phosphate buffer (0.1 M, pH 2, 11, and 12), citrate buffer (0.1 M, pH 3–7), and borate buffer (0.1 M, pH 8–10). Ultrapure water (18 MΩ·cm, Milli-Q^®^ UF Plus, Millipore, Bedford, MA, USA) was used throughout.

### 3.3. Preparation and Measurement Procedure

#### 3.3.1. Standard Solutions

A stock solution of GFB (5 g/L) was prepared in dimethyl sulfoxide (DMSO)/acetonitrile (1:4). A 100-fold dilution of this stock was diluted with acetonitrile to give a 50 µg/mL working solution, used to prepare concentrations from 50 to 1200 ng/mL for the calibration range. A second working solution (10 µg/mL) was used to prepare concentrations from 50 to 300 ng/mL.

DMSO was required to dissolve gefitinib at 5 g L^−1^, and the subsequent dilutions were made with acetonitrile. The volume of organic solution carried into the final measurement flask does not exceed about 0.12 mL in a 5 mL final volume (not more than approximately 2.4% *v*/*v*); the consequences of this organic-solvent footprint for the greenness assessment are discussed in Section 2.6.

#### 3.3.2. Tablet Sample Solution

Ten Iressa^®^ tablets, each labeled to contain 250 mg of GFB, were accurately weighed and finely powdered. An accurately weighed amount of the powdered tablets equivalent to 100 mg of GFB was transferred into a 50 mL volumetric flask. Approximately 5 mL of DMSO–acetonitrile (1:4, *v*/*v*) was added, and the mixture was sonicated for 10 min, followed by shaking for 15 min to ensure complete extraction of GFB. The volume was then completed to the mark with acetonitrile and mixed well.

The resulting solution was filtered through a suitable filter, and the first portion of the filtrate was discarded. Appropriate serial dilutions of the filtrate were then prepared with acetonitrile to obtain tablet sample solutions suitable for the proposed spectrofluorimetric procedure. The final measured tablet solutions were prepared according to the method in Section 2.4, within the validated calibration range of 50–1200 ng mL^−1^, particularly at 100, 250, and 500 ng mL^−1^ for recovery and application studies.

#### 3.3.3. Construction of the Calibration Graph

Aliquots of standard or tablet sample solutions were transferred into a series of 5 mL volumetric flasks to obtain final GFB concentrations within the range of 50–1200 ng mL^−1^. To each flask, 0.25 mL of 0.05% *w*/*v* Cremophor RH 40 solution was added, giving a final Cremophor RH 40 concentration of 0.005% *w*/*v*, equivalent to approximately 0.02 mM based on an average molecular weight of about 2500 g mol^−1^. Then, 1.0 mL of borate buffer pH 8.0 was added, and the solutions were diluted to volume with distilled water.

The contents of each flask were mixed thoroughly and allowed to stand for 5 min at room temperature to ensure equilibration of GFB within the micellar medium before measurement. Fluorescence intensity was measured at an emission wavelength of 394 nm following excitation at 333 nm, using the optimized instrumental conditions. The relative fluorescence intensity (RFI) values were plotted against the corresponding GFB concentrations, and the calibration data were processed by least-squares linear regression (Table 5).

The calibration graph was constructed by plotting the RFI values against the corresponding GFB concentrations (ng mL^−1^). The obtained data were subjected to least-squares linear regression analysis using a weighting factor of 1. A strong linear relationship was observed between the RFI values and GFB concentrations over the investigated concentration range of 50–1200 ng mL^−1^, with a correlation coefficient close to unity (Figure 11). The regression parameters are summarized in Table 6. The limits of detection (LOD) and limits of quantification (LOQ) were estimated according to ICH Q2(R2) guidelines. The LOD was defined as the lowest concentration of GFB that could be reliably detected, whereas the LOQ was defined as the lowest concentration that could be quantified with acceptable accuracy and precision. These parameters were calculated using the following equations:

LOD = 3.3 × S_a_/b

LOQ = 10 × S_a_/b

where S_a_ is the standard deviation of the intercept and b is the slope of the regression line. The calculated LOD and LOQ values for GFB are presented in Table 6. As explained in Section 2.3.2, the calculated LOQ was not adopted as the lower limit of the validated range because accuracy and precision were not determined at that concentration; 50 ng mL^−1^, the lowest level at which they were determined, was retained instead.

### 3.4. Computational Methods

Computational calculations were performed to provide a qualitative electronic interpretation of the excitation and emission-related behavior of GFB. The molecular structure of GFB was built and visualized using GaussView 6.0, and all quantum-chemical calculations were carried out using Gaussian 09W [39]. Geometry optimization was performed prior to the excited-state calculations, and the optimized structure was used for subsequent TD-DFT analysis.

The electronic excitation spectrum was calculated using the time-dependent self-consistent field density functional theory approach (TD-SCF/DFT) with the long-range corrected CAM-B3LYP functional and the 6-311G(d,p) basis set [40]. The excitation calculation was performed for neutral GFB with charge = 0 and singlet spin state. Twenty singlet excited states were calculated to identify the electronic transitions contributing to the experimental excitation region. The calculated excitation transitions, oscillator strengths, and molecular orbital contributions were analyzed to support the interpretation of the experimental excitation wavelengths.

Triplet-state excited-state calculations were performed using unrestricted time-dependent density functional theory at the UCAM-B3LYP/6-311G(d,p) level [40]. Solvent effects were represented using the integral-equation-formalism polarizable continuum model, IEF-PCM, for chloroform, acetonitrile, ethanol, methanol, and water, while an additional calculation was performed in the gas phase as an isolated-molecule reference. No explicit solvent molecules, counterions, or surfactant molecules were included, and the Cremophor RH 40 micellar pseudophase was not represented computationally. The neutral molecule was assigned a charge of 0 and a triplet multiplicity of 3. Twenty excited states were calculated, and the first root was selected for detailed analysis. The resulting transition energies and orbital contributions were used only as qualitative descriptors of the low-energy triplet-state electronic structure and orbital redistribution. Because these calculations were based on a triplet-state treatment and did not constitute an optimized singlet excited-state S_1_ → *S*_0_ emission calculation, the resulting wavelengths were not interpreted as quantitative predictions of the experimental fluorescence maxima, fluorescence intensities, or quantum yields.

The simulated excitation and emission-related spectra were processed using GaussSum [41], while molecular orbital compositions, orbital visualization, and electronic-transition analysis were supported by Multiwfn [42]. Frontier molecular orbitals, including the highest occupied molecular orbital (HOMO), lowest unoccupied molecular orbital (LUMO), and nearby occupied and virtual orbitals, were examined to identify the main orbital contributions involved in GFB excitation and emission-related transitions.

The computational results were interpreted qualitatively. The calculated excitation transition at 326.99 nm, mainly assigned to the HOMO → LUMO transition and lying at lower energy than the principal calculated band near 292 nm discussed in Section 2.1.1, was used only to support the electronic feasibility of GFB excitation in the region of the experimental micellar excitation wavelength. In contrast, the micellar fluorescence enhancement observed experimentally in Cremophor RH 40 was attributed mainly to micellar solubilization, protection of GFB from aqueous quenching, and restriction of non-radiative relaxation, rather than to oscillator strength alone. The limits of this computational component are stated explicitly. The calculated excitation energies reproduce the experimental excitation maxima to within about 10 nm, but the emission-related values, which derive from a triplet-spin configuration, are 39–123 nm (0.18–0.69 eV) longer than the measured fluorescence maxima and do not reproduce the micellar emission at 394 nm. The calculations are therefore used exclusively for the qualitative assignment of the orbitals and of the type of transition involved, and are not used to predict, fit or validate fluorescence wavelengths, quantum yields or intensities; no quantitative photophysical conclusion in this work rests on them.

## 4. Conclusions

A micelle-enhanced spectrofluorimetric method using Cremophor RH 40 was developed for the average-content assay of intact gefitinib tablets based on native fluorescence measured at λ_ex_/λ_em_ = 333/394 nm. The method was validated over the concentration range of 50–1200 ng mL^−1^. The calculated LOD and LOQ were 6.89 and 20.89 ng mL^−1^, respectively; however, the calculated LOQ was not experimentally verified for accuracy and precision and therefore does not represent a validated quantification limit. Response-surface analysis supported the local suitability of the selected operating conditions but should not be interpreted as full predictive validation or as establishing an analytical procedure design space.

The method showed adequate selectivity for gefitinib in the investigated intact tablet matrix following the specified extraction procedure. However, degradation products and process-related impurities were not specifically evaluated; therefore, the method has not been demonstrated to be stability-indicating or suitable for impurity determination. Its validated application should be restricted to the average-content assay of intact gefitinib tablets. Because the tablets were analyzed as a composite powder, the reported assay does not represent an assessment of content uniformity. The between-day precision result at 500 ng mL^−1^ did not meet the predefined acceptance criterion, and intermediate precision in the mid-range should therefore be re-established before routine use. Buffer pH must also be strictly controlled around 8.0 because recovery at pH 8.3 approached or slightly exceeded the predefined upper limit.

The TD-DFT calculations provided qualitative support for the electronic-transition assignments, while the AGREE assessment indicated a comparatively favorable environmental profile, although acetonitrile and DMSO remained necessary during sample preparation. Comparison with the reference spectrofluorimetric method is descriptive only because no matched-sample evaluation under identical experimental conditions was performed; consequently, no statistical equivalence or analytical superiority is claimed. Overall, the method may be applied to the average-content assay of intact gefitinib tablets within its validated range, subject to strict pH control and successful re-establishment of mid-range intermediate precision.

## Figures and Tables

**Table 1:** Relative fluorescence intensity (RFI) of gefitinib in different solvents (0.5 µg/mL) compared with the micellar Cremophor RH 40 reference system (1 µg/mL, pH 8 borate buffer).

Solvent/Medium	λ_EX_ (nm)		RFI at λ_EX_	λ_EM_ (nm)		RFI at λ_EM_	Comment
	EXP	DFT		EXP	DFT		
Chloroform	286	292.45	7524.29	488	537.85	7547.22	Highest fluorescence response among neat solvents
Acetonitrile	282	292.5	1122.36	449	534.88	1744.25	Excitation recorded for EM around 449 nm
Acetonitrile	282	292.5	1751.34	496		1744.25	Excitation recorded for EM around 496 nm
Methanol	288	292.38	268.98	418	534.39	268.16	Weak blue-shifted emission
Ethanol	284	292.56	514.97	412	535.44	529.8	Moderate emission with blue-shifted λ_EM_
Water	290	292.52	186.04	444	534.51	185.53	Weak aqueous fluorescence
GAS		286.6		519.41	547.49		
Micellar Cr RH 40, buffer, pH 8	333			394		1486.75	

**Table 2:** Calculated dipole moment of GFB in the gas phase and in selected solvents, together with the corresponding static dielectric constants (ε_s_).

Solvent	Dipole Moment (Debye)	Dielectric Constant (ε_s_)
Ethanol	7.987894	24.3
Gas	5.40477	
Chloroform	7.169632	4.7
Acetonitrile	8.05979	36
Methanol	8.0456	33
Water	5.40477	78.4

**Table 3:** Critical micelle concentration (CMC) and working concentration of the organized media screened.

Surfactant	Approximate CMC	Working Concentration (mM)
Tween 20 (Polysorbate 20)	0.05–0.10 mM	0.10
Tween 80 (Polysorbate 80)	0.012–0.015 mM	0.02
Tween 85 (Polysorbate 85)	0.014–0.015 mM	0.016
Cremophor EL	0.012–0.03 mM	0.03
Cremophor RH 40	0.016–0.018 mM	0.02
HCO-30	0.09–0.13 mM	0.15
HCO-40	0.016–0.018 mM	0.04
HCO-60	0.06–0.08 mM	0.08
SDS (Sodium dodecyl sulfate)	8.0–8.5 mM	8.0

**Table 4:** Analysis of variance (ANOVA) for the full quadratic response-surface model describing the effects of buffer volume (A), Cremophor RH 40 concentration (B), and buffer pH (C) on the relative fluorescence intensity (RFI) of the GFB–Cremophor RH 40 system.

Source	Sum of Squares	df	Mean Square	F-Value	*p*-Value	Remark
Model	1.28 × 10^+5^	9	14,244.27	83.26	0.0019	significant
A-Buffer Volume	512	1	512	2.99	0.1821	
B-surfactant Conc.	1.13	1	1.13	0.0066	0.9405	
C-pH	9045.13	1	9045.13	52.87	0.0054	
AB	36	1	36	0.2104	0.6776	
AC	1089	1	1089	6.37	0.086	
BC	6972.25	1	6972.25	40.75	0.0078	
A^2^	27,720.04	1	27,720.04	162.03	0.001	significant
B^2^	18,054.32	1	18,054.32	105.53	0.002	
C^2^	1.10 × 10^+5^	1	1.10 × 10^5^	640.04	0.0001	significant
Residual	513.25	3	171.08			
Cor Total	1.29 × 10^+5^	12				

**Table 5:** Experimental verification of the response-surface model at the selected analytical conditions.

Experiment	Predicted RFI	Observed RFI	Prediction Error (%)
1	1205	1193.5	0.954
2	1205	1216.01	0.914
3	1205	1198.8	0.515
Mean	1205	1202.77	0.794

**Table 6:** Analytical performance data for the spectrofluorimetric determination of GFB.

Parameter	Value
Wavelength [λ_ex_/λ_em_] (nm)	333/394
Linearity range (ng/mL)	50–1200
Intercept (a)	20.99
Slope (b)	1.483
Correlation coefficient (r)	0.9997
S.D. of residuals (*S_y_*_/*x*_)	10.43
S.D. of intercept (*S_a_*)	±3.098
S.D. of slope (*S_b_*)	±0.0051
Equation	*Y* = 1.483 × *x* + 20.99
% RSD	0.34
% Error	0.343
LOD (ng/mL)	6.89
LOQ (ng/mL)	20.89
Number of calibration points (*n*)	6

**Table 7:** Accuracy and precision data for the determination of GFB by the proposed spectrofluorimetric method.

Amount Taken (ng/mL)	Number of Independent Preparations	%Found ± SD	%RSD	%Error	Assessment
200 (intra-day)	6	100.52 ± 1.53	1.52	0.52	Meets
500 (intra-day)	6	97.55 ± 0.32	0.33	2.45	Meets
1000 (intra-day)	6	101.32 ± 1.32	1.30	1.32	Meets
200 (inter-day)	6	102.22 ± 1.65	1.61	2.22	Meets
500 (inter-day)	6	99.18 ± 3.21	3.24	0.82	Does not meet
1000 (inter-day)	6	101.21 ± 1.82	1.80	1.21	Meets

Each value was obtained from six independently prepared solutions (*n* = 6, *n* = 6, *n* = 6). Each preparation was processed and measured separately through the complete analytical procedure; repeated instrumental readings of the same solution were not treated as independent replicates. “Meets” and “Does not meet” refer to the predefined acceptance criteria of a mean recovery within 95.0–105.0% and a %RSD of not more than 3.0%. The inter-day result at 500 ng mL^−1^, with a %RSD of 3.24%, was the only result outside these predefined limits. This result was retained without re-testing or reclassification and is discussed in the preceding text and in Section 2.7.

**Table 8:** Robustness of the proposed spectrofluorimetric method to small deliberate changes in experimental parameters.

Parameter Variation	Recovery (%) ± SD	%RSD	Absolute Difference from Nominal Recovery *	Assessment
No variation (nominal conditions)	102.4 ± 0.25	0.24	0	Meets
Cremophor RH 40 volume: 950 µL	97.12 ± 1.22	1.26	5.28	Meets
Cremophor RH 40 volume: 1050 µL	98.95 ± 2.10	2.12	3.45	Meets
pH 7.7	98.17 ± 1.37	1.4	4.23	Meets
pH 8.3	105.05 ± 2.31	2.2	2.65	At or slightly above the upper limit
Buffer volume: 950 µL	98.25 ± 2.69	2.74	4.15	Meets
Buffer volume: 1050 µL	101.15 ± 2.31	2.28	1.25	

* The absolute difference was calculated as the absolute difference, in percentage points, between the mean recovery under each modified condition and the mean recovery under the nominal conditions.

**Table 9:** Determination of gefitinib in independently prepared bulk-standard and tablet-sample solutions using the proposed spectrofluorimetric method.

Bulk Powder			Tablets		
Taken Conc.	Found Conc.	Recovery (%)	Nominal Tablet-Sample Concentration (ng mL^−1^)	Measured Concentration (ng mL^−1^)	Percentage Found (%)
(ng mL^−1^)	(ng mL^−1^)		(ng mL^−1^)	(ng mL^−1^)	
100	98.98 ± 0.58	98.98 ± 0.58	100	100.42 ± 1.02	100.42 ± 1.02
250	252.80 ± 2.80	101.12 ± 1.12	250	245.65 ± 2.23	98.26 ± 0.89
500	495.70 ± 2.90	99.14 ± 0.58	500	509.80 ± 4.85	101.96 ± 0.97
Mean	—	99.75	Mean	—	100.21
±SD	—	±1.19	±SD	—	±1.86

Each result represents 6 independently prepared analytical solutions. The tablet-sample concentrations were prepared by dilution of the composite tablet extract and were not obtained by adding a reference standard to an existing tablet-sample solution. Percentage found was calculated as (measured concentration/nominal concentration) × 100.

**Table 10:** Comparison of the key analytical parameters of the proposed micelle-enhanced spectrofluorimetric method with those of the only previously reported spectrofluorimetric method for gefitinib [7].

Parameter	Proposed Method	Reported Method [7]
Analytical principle	Native fluorescence enhanced by Cremophor RH 40	Native fluorescence in ethanol
Medium of final measurement	Aqueous 0.1 M borate buffer, pH 8.0, containing 0.005% *w*/*v* Cremophor RH 40.	Ethanol
λ_ex_/λ_em_ (nm)	333/394	280/512
Linear range	50–1200 ng mL^−1^ (six levels)	1000–3500 ng mL^−1^; six levels)
Correlation coefficient (r)	0.9997	0.9995
LOD (ng mL^−1^)	6.89	29.9 ng mL^−1^
LOQ (ng mL^−1^)	20.89 (calculated); 50 adopted as the lowest validated level (Section 2.3.2)	90.6 ng mL^−1^
Intra-day precision (%RSD)	0.33–1.52 (*n* = 6 per level)	0.3572% (*n* = 6)
Inter-day precision (%RSD)	1.61–3.24 (*n* = 6 per level, 3 days)	0.2565% (*n* = 6)
Derivatization	Not required	Not required
Optimization strategy	Univariate screening followed by Box–Behnken response-surface confirmation	Univariate only
Mechanistic/computational support	DFT and TD-DFT (singlet and triplet), frontier-orbital analysis in six media	None reported
Tablet assay (% of label claim)	100.21 ± 1.86 (Iressa^®^ 250 mg, composite of 10 tablets)	100.47 ± 0.42% (*n* = 6; Geftistar 250 mg tablets)
Excipient/matrix evaluation	Placebo blend plus recovery from the authentic formulation (Table 9)	Standard-addition recovery in the tablet matrix: 99.33–101.61%; mean recovery 100.41 ± 0.59% (*n* = 9)
Organic solvent in the measured solution	≤0.12 mL per 5 mL final volume (≤2.4% *v*/*v*)	The whole measurement volume is organic solvent
Greenness assessment	AGREE score 0.61	Score 0.46; ethanol is, however, a greener solvent than acetonitrile (Section 2.6)
Head-to-head statistical comparison	Not performed; see text	Not performed; see text

Values for the proposed method are taken from Table 6, Table 7, Table 8 and Table 9 of this work. Values for the reported method are taken from the original publication. The two datasets were generated in different laboratories, on different samples and with different replicate designs; accordingly, no t-test, F-test or ANOVA has been applied between them and no claim of statistical superiority or equivalence is made (Section 2.5).

## Data Availability

The datasets generated and/or analyzed during the current study are available from the corresponding author upon reasonable request.

## Supplementary Materials

The following supporting information can be downloaded at: https://www.mdpi.com/article/10.3390/ijms27209015/s1.

## Abbreviations

The following abbreviations are used in this manuscript:

AGREE	Analytical GREEnness Metric Approach
ANOVA	Analysis of Variance
BBD	Box–Behnken Design
CAM-B3LYP	Coulomb-Attenuating Method Becke Three-Parameter Lee–Yang–Parr Functional
CMC	Critical Micelle Concentration
CV	Coefficient of Variation
DFT	Density Functional Theory
DMSO	Dimethyl Sulfoxide
EGFR	Epidermal Growth Factor Receptor
FDA	United States Food and Drug Administration
GAC	Green Analytical Chemistry
GFB	Gefitinib
HOMO	Highest Occupied Molecular Orbital
HPLC	High-Performance Liquid Chromatography
HPTLC	High-Performance Thin-Layer Chromatography
ICH	International Council for Harmonisation
IEF-PCM	Integral-Equation-Formalism Polarizable Continuum Model
LC-MS	Liquid Chromatography–Mass Spectrometry
LOD	Limit of Detection
LOQ	Limit of Quantification
LUMO	Lowest Unoccupied Molecular Orbital
NSCLC	Non-Small-Cell Lung Cancer
OFAT	One-Factor-at-a-Time
OPTN	Optineurin
PRESS	Predicted Residual Error Sum of Squares
RFI	Relative Fluorescence Intensity
RP-HPLC	Reversed-Phase High-Performance Liquid Chromatography
RSD	Relative Standard Deviation
RSM	Response-Surface Methodology
SD	Standard Deviation
SDS	Sodium Dodecyl Sulfate
TD-DFT	Time-Dependent Density Functional Theory
TKI	Tyrosine Kinase Inhibitor
UPLC	Ultra-Performance Liquid Chromatography
