# Supplementary material for: Green Micelle-Enhanced Spectrofluorimetric Determination of Gefitinib in Pharmaceutical Tablets Using Cremophor RH 40: Response-Surface Optimization and Time-Dependent Density Functional Theory (TD-DFT) Interpretation
Source: Int J Mol Sci. 2026 Oct 11;27(20):9015. doi: 10.3390/ijms27209015 (PMC13704709; doi:10.3390/ijms27209015)
Supplement: Supplementary file 1 [file ijms-27-09015-s001.zip › ijms-4583341-supplementary.pdf]

Table S1. Important TD-DFT excitation transitions of GFB from EXData.txt

**State**	**Energy (cm^{-1})**	**Wavelength (nm)**	**Osc. Strength**	**Symmetry / Spin**	**Major Electronic Contributions**
			**Water**		
6	34186	292.52	0.6515	1.000-A	HOMO(A)→LUMO(A) 45%, HOMO(B)→LUMO(B) 45%
10	38390	260.48	0.0806	1.000-A	H−2(A/B)→LUMO(A/B) 11% each; HOMO(A/B)→L+1(A/B) 26% each
12	39654	252.18	0.0386	1.000-A	HOMO(A)→L+2(A) 22%, HOMO(B)→L+2(B) 22%
14	41588	240.45	0.342	1.000-A	H−3(A)→LUMO(A) 26%, H−3(B)→LUMO(B) 26%
18	44024	227.15	0.5191	1.000-A	H−2(A/B)→LUMO(A/B) 15% each; HOMO(A/B)→L+2(A/B) 15% each
			Gas		
7	34890	286.62	0.5047	1.000-A	HOMO(A)→LUMO(A) 42%, HOMO(B)→LUMO(B) 42%
10	38692	258.45	0.0747	1.000-A	HOMO(A/B)→L+1(A/B) 28% each
13	39781	251.38	0.0442	1.000-A	HOMO(A/B)→L+2(A/B) 24% each
15	42317	236.31	0.2899	1.000-A	H−3(A/B)→LUMO(A/B) 24% each
			Acetonitrile		
1	34188	292.5	0.6535	Singlet-A	HOMO → LUMO (89%)
5	41596	240.41	0.3412	Singlet-A	H−3 → LUMO (53%), HOMO → L+3 (14%)
6	44017	227.19	0.5246	Singlet-A	H−3 → LUMO (18%), H−2 → LUMO (30%), HOMO → L+1 (14%), HOMO → L+2 (30%)
17	53655	186.38	0.5541	Singlet-A	H−4 → LUMO (12%), H−3 → L+2 (12%), H−2 → L+2 (11%), H−2 → L+3 (14%), HOMO → L+6 (21%)
			Chloroform		
1	34226	292.17	0.6669	Singlet-A	HOMO → LUMO (89%)
5	41686	239.89	0.3374	Singlet-A	H−3 → LUMO (54%), HOMO → L+3 (15%)
6	43975	227.4	0.5711	Singlet-A	H−3 → LUMO (15%), H−2 → LUMO (32%), HOMO → L+1 (13%), HOMO → L+2 (32%)
9	47928	208.65	0.418	Singlet-A	H−2 → L+1 (25%), H−2 → L+2 (26%), HOMO → L+3 (11%)

17	53585	186.62	0.5629	Singlet-A	H−3 → L+2 (13%), H−2 → L+2 (10%), H−2 → L+3 (16%), HOMO → L+6 (23%)

Table S2. Important TD-DFT emission-related transitions of GFB from EMData.txt

No.	Energy (cm-1)	Wavelength (nm)	Osc. Strength	Symmetry	Major contribs	Minor contribs
Water						
5	18708.8	534.5	0.3923	3.041-A	HOMO(A)->L+1(A) (12%), HOMO(A)->L+2(A) (21%), HOMO(B)->LUMO(B) (42%)	HOMO(A)->L+3(A) (8%), H-5(B)->LUMO(B) (2%), H-4(B)->LUMO(B) (2%), H-3(B)->LUMO(B) (4%), HOMO(B)->L+1(B) (2%)
7	24845.1	402.5	0.1246	3.295-A	HOMO(A)->L+2(A) (16%), HOMO(A)->L+3(A) (19%), H-6(B)->LUMO(B) (26%)	H-1(A)->LUMO(A) (2%), H-1(A)->L+1(A) (2%), H-1(A)->L+2(A) (2%), H-1(A)->L+3(A) (3%), HOMO(A)->L+1(A) (6%), H-9(B)->LUMO(B) (2%), HOMO(B)->L+1(B) (4%), HOMO(B)->L+4(B) (3%)
10	29336.0	340.9	0.0698	3.456-A	H-9(B)->LUMO(B) (11%), H-6(B)->LUMO(B) (19%)	H-7(A)->L+1(A) (3%), H-5(A)->LUMO(A) (2%), H-5(A)->L+1(A) (2%), H-1(A)->L+2(A) (3%), H-1(A)->L+3(A) (3%), HOMO(A)->L+3(A) (7%), HOMO(A)->L+11(A) (2%), H-5(B)->LUMO(B) (3%), H-4(B)->LUMO(B) (4%), H-4(B)->L+2(B) (3%), H-3(B)->LUMO(B) (5%), H-3(B)->L+2(B) (3%), HOMO(B)->LUMO(B) (3%), HOMO(B)->L+1(B) (3%), HOMO(B)->L+4(B) (5%)
Gas						
5	19252.5	519.4	0.3485	3.044-A	HOMO(A)->L+1(A) (17%), HOMO(A)->L+2(A) (15%), HOMO(B)->LUMO(B) (47%)	HOMO(A)->L+4(A) (8%), H-10(B)->LUMO(B) (2%), HOMO(B)->L+1(B) (2%)
7	25232.2	396.3	0.1184	3.298-A	HOMO(A)->L+1(A) (13%), HOMO(A)->L+2(A) (10%), HOMO(A)->L+4(A) (11%), H-6(B)->LUMO(B) (29%)	H-1(A)->L+1(A) (4%), H-1(A)->L+4(A) (3%), HOMO(A)->L+3(A) (3%), H-10(B)->LUMO(B) (2%), H-7(B)->LUMO(B) (3%), H-4(B)->L+2(B) (2%), HOMO(B)->L+1(B) (4%), HOMO(B)->L+4(B) (3%)
16	36149.0	276.6	0.1007	3.469-A	H-1(B)->L+1(B) (22%)	H-4(A)->LUMO(A) (2%), H-4(A)->L+2(A) (3%), H-3(A)->L+2(A) (7%), HOMO(A)->L+12(A) (3%), HOMO(A)->L+13(A) (6%), HOMO(A)->L+14(A) (3%), HOMO(A)->L+15(A) (2%), H-12(B)->LUMO(B) (5%), H-10(B)->LUMO(B) (2%), H-6(B)->L+1(B) (3%), H-1(B)->L+3(B) (7%), H-1(B)->L+4(B) (5%), HOMO(B)->L+1(B) (3%)
Acetonitrile						

5	18695.9	534.9	0.3949	3.041-A	HOMO(A)->L+1(A) (12%), HOMO(A)->L+2(A) (21%), HOMO(B)->LUMO(B) (43%)	HOMO(A)->L+3(A) (8%), H-4(B)->LUMO(B) (3%), H-3(B)->LUMO(B) (4%), HOMO(B)->L+1(B) (2%)
7	24848.3	402.4	0.1246	3.295-A	HOMO(A)->L+2(A) (15%), HOMO(A)->L+3(A) (19%), H-6(B)->LUMO(B) (27%)	H-1(A)->LUMO(A) (2%), H-1(A)->L+1(A) (2%), H-1(A)->L+2(A) (2%), H-1(A)->L+3(A) (3%), HOMO(A)->L+1(A) (6%), H-9(B)->LUMO(B) (2%), HOMO(B)->L+1(B) (4%), HOMO(B)->L+4(B) (3%)
10	29330.3	340.9	0.0693	3.460-A	H-9(B)->LUMO(B) (11%), H-6(B)->LUMO(B) (18%)	H-7(A)->L+1(A) (3%), H-5(A)->LUMO(A) (2%), H-5(A)->L+1(A) (2%), H-1(A)->L+2(A) (3%), H-1(A)->L+3(A) (3%), HOMO(A)->L+3(A) (7%), HOMO(A)->L+11(A) (2%), H-5(B)->LUMO(B) (2%), H-4(B)->LUMO(B) (6%), H-4(B)->L+2(B) (3%), H-3(B)->LUMO(B) (5%), H-3(B)->L+2(B) (3%), HOMO(B)->LUMO(B) (3%), HOMO(B)->L+1(B) (3%), HOMO(B)->L+4(B) (5%)
Chloroform						
**5**	**18593**	**537.9**	**0.4129**	**3.040-A**	**HOMO(A)→L+1(A) (14%), HOMO(A)→L+2(A) (21%), HOMO(B)→LUMO(B) (44%)**	HOMO(A)→L+3(A) (8%), H−4(B)→LUMO(B) (3%), H−3(B)→LUMO(B) (3%), HOMO(B)→L+1(B) (2%)
**7**	**24879**	**402.0**	**0.1255**	**3.294-A**	**HOMO(A)→L+2(A) (14%), HOMO(A)→L+3(A) (18%), H−6(B)→LUMO(B) (28%)**	H−1(A)→L+1(A) (3%), H−1(A)→L+2(A) (2%), H−1(A)→L+3(A) (3%), HOMO(A)→L+1(A) (8%), H−9(B)→LUMO(B) (2%), H−4(B)→L+2(B) (2%), HOMO(B)→L+1(B) (4%), HOMO(B)→L+4(B) (3%)
20	**35813**	**279.2**	**0.1306**	**3.432-A**	**HOMO(A)→L+12(A) (15%), H−2(B)→L+1(B) (19%)**	H−4(A)→LUMO(A) (2%), H−3(A)→L+2(A) (7%), HOMO(A)→L+4(A) (2%), HOMO(A)→L+13(A) (3%), H−13(B)→LUMO(B) (2%), H−12(B)→LUMO(B) (4%), H−9(B)→LUMO(B) (3%), H−6(B)→L+1(B) (3%), H−2(B)→L+3(B) (5%), H−2(B)→L+4(B) (5%)

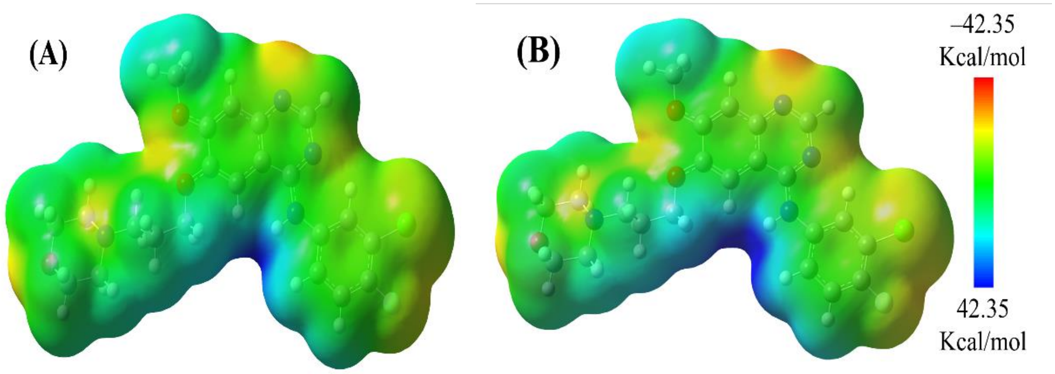

Figure S1. TD-DFT electrostatic potential maps of gefitinib showing charge distribution in the (A) singlet and (B) triplet states. The maps indicate broad charge delocalization over the quinazoline–anilino framework, with slightly stronger polarization in the triplet state.

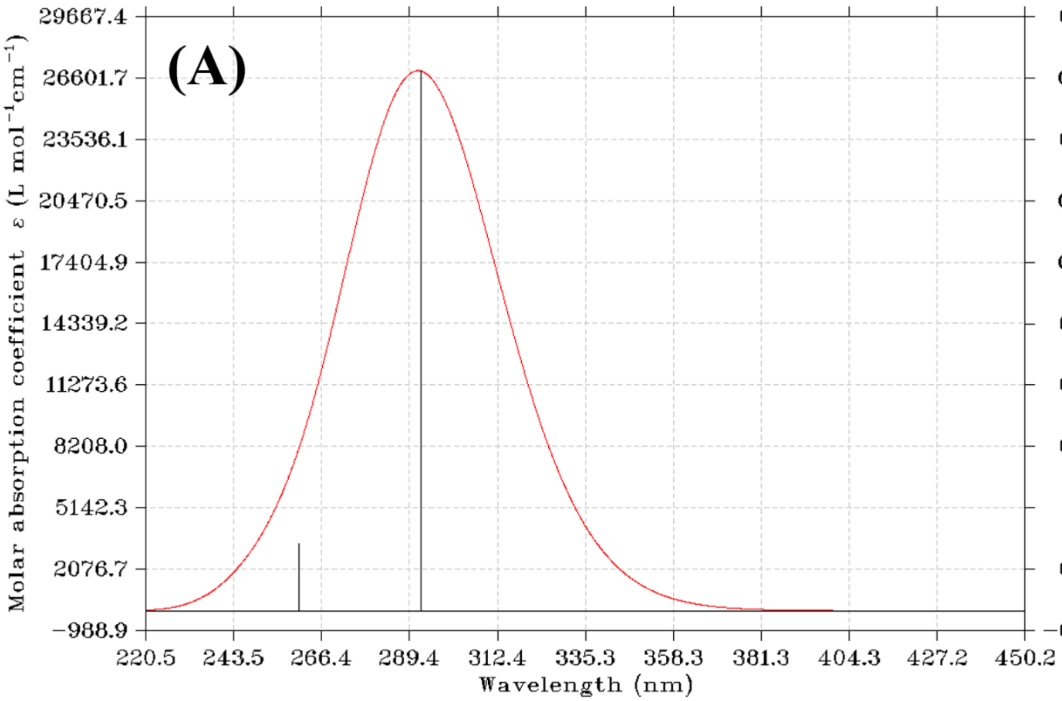

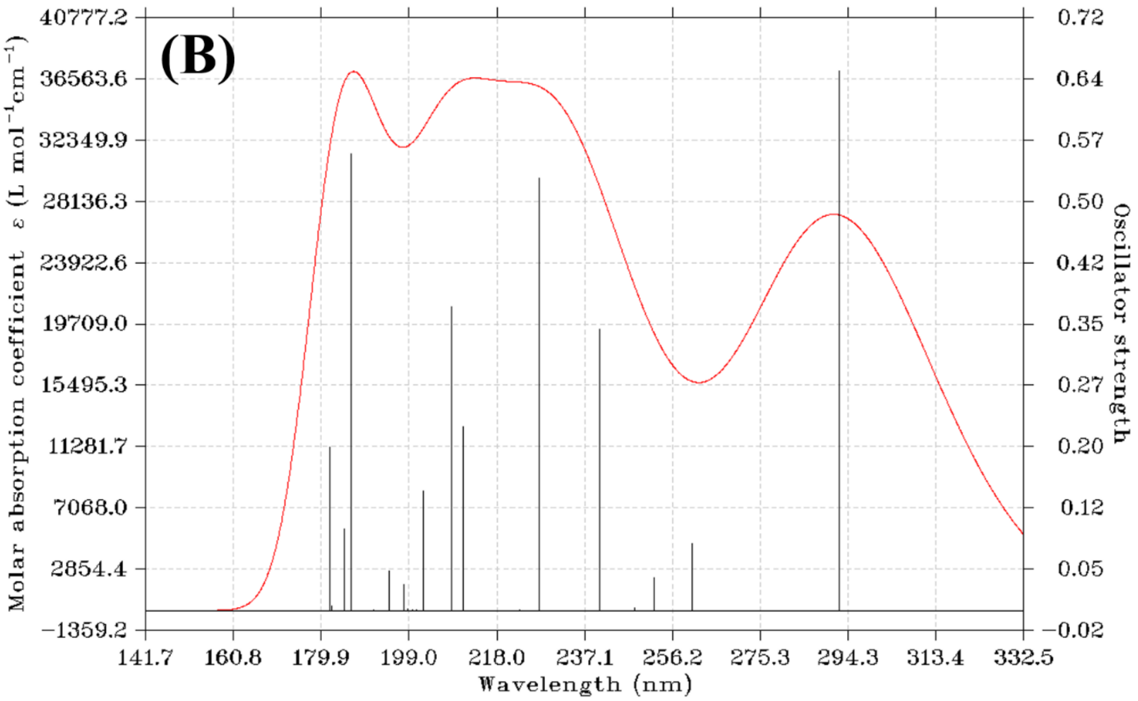
(B)
Molar absorption coefficient ε (L mol⁻¹cm⁻¹)
40777.2
36563.6
32349.9
28136.3
23922.6
19709.0
15495.3
11281.7
7068.0
2854.4
−1359.2
Oscillator strength
0.72
0.64
0.57
0.50
0.42
0.35
0.27
0.20
0.12
0.05
−0.02
141.7
160.8
179.9
199.0
218.0
237.1
256.2
275.3
294.3
313.4
332.5
Wavelength (nm)

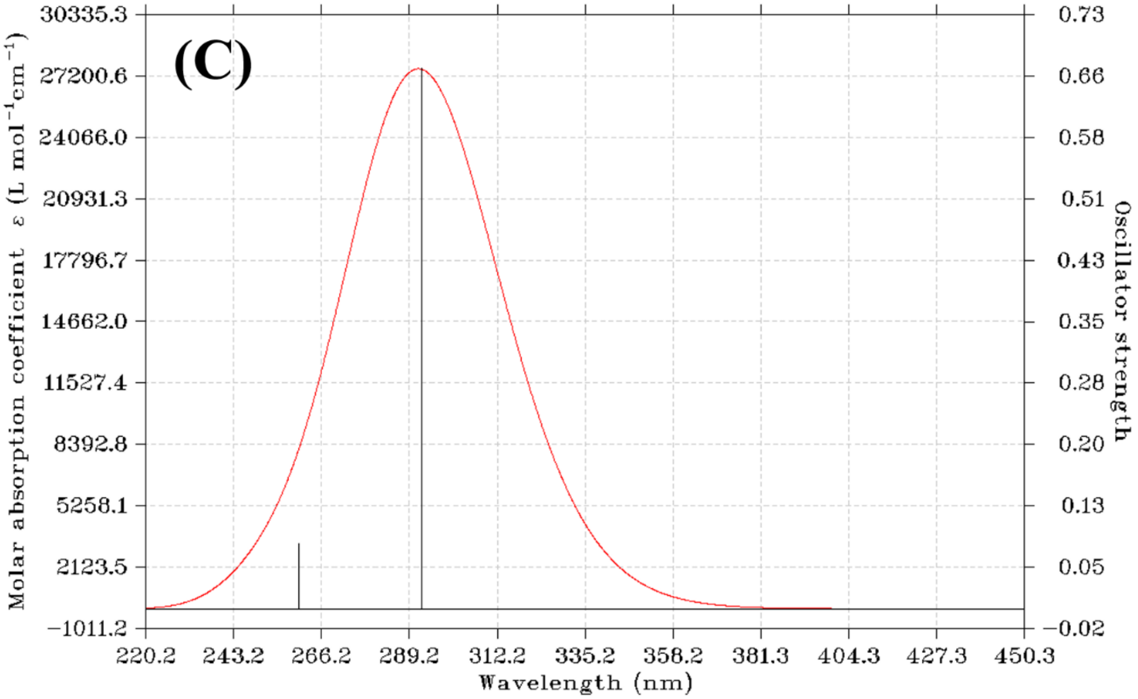
(C)
Molar absorption coefficient ε (L mol⁻¹cm⁻¹)
30335.3
27200.6
24066.0
20931.3
17796.7
14662.0
11527.4
8392.8
5258.1
2123.5
−1011.2
Oscillator strength
0.73
0.66
0.58
0.51
0.43
0.35
0.28
0.20
0.13
0.05
−0.02
220.2
243.2
266.2
289.2
312.2
335.2
358.2
381.3
404.3
427.3
450.3
Wavelength (nm)

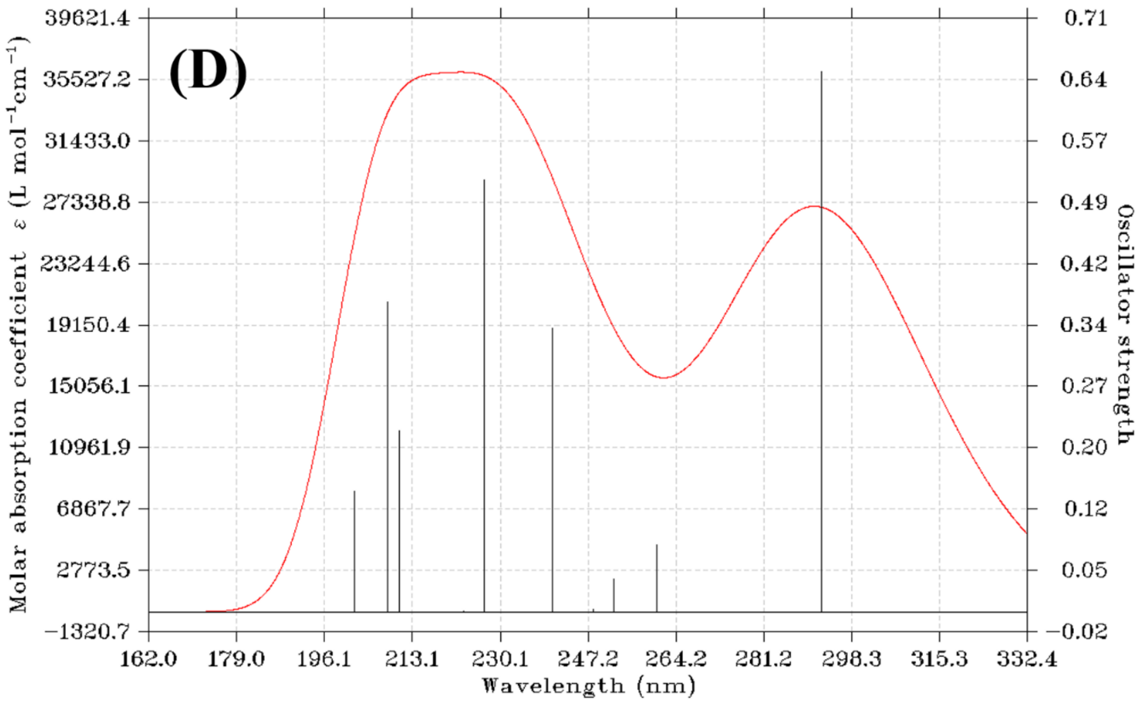
(D)
Molar absorption coefficient ε (L mol⁻¹cm⁻¹)
Oscillator strength
Wavelength (nm)
39621.4
35527.2
31433.0
27338.8
23244.6
19150.4
15056.1
10961.9
6867.7
2773.5
−1320.7
0.71
0.64
0.57
0.49
0.42
0.34
0.27
0.20
0.12
0.05
−0.02
162.0
179.0
196.1
213.1
230.1
247.2
264.2
281.2
298.3
315.3
332.4

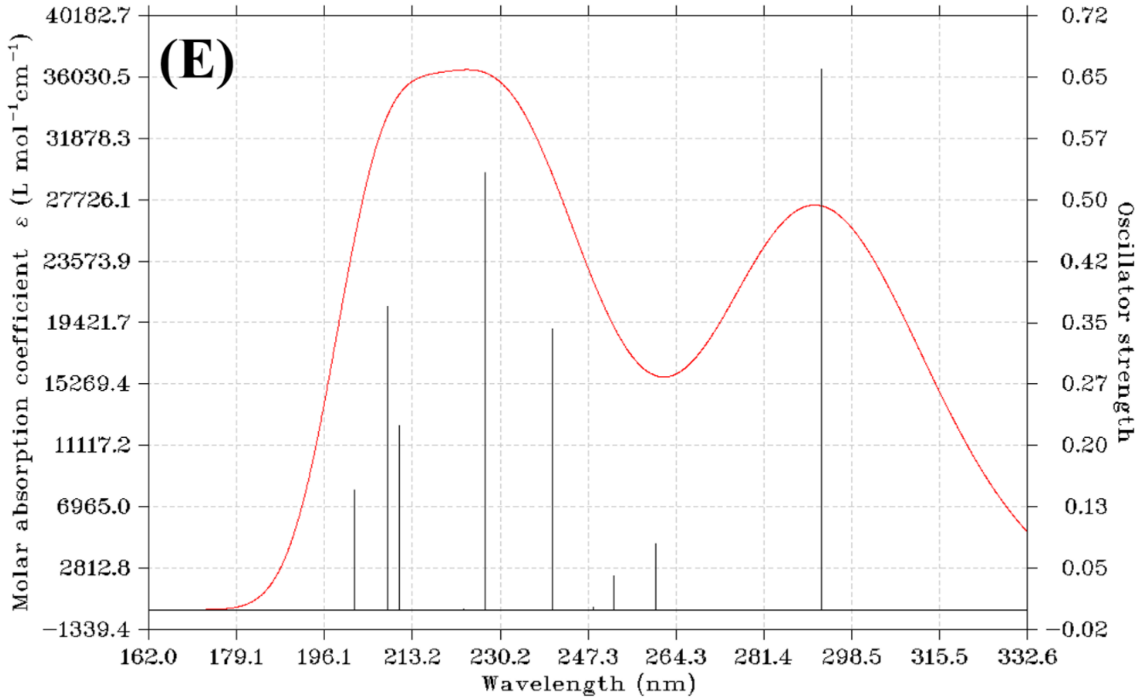
(E)
Molar absorption coefficient ε (L mol⁻¹cm⁻¹)
Oscillator strength
Wavelength (nm)
40182.7
36030.5
31878.3
27726.1
23573.9
19421.7
15269.4
11117.2
6965.0
2812.8
−1339.4
0.72
0.65
0.57
0.50
0.42
0.35
0.27
0.20
0.13
0.05
−0.02
162.0
179.1
196.1
213.2
230.2
247.3
264.3
281.4
298.5
315.5
332.6

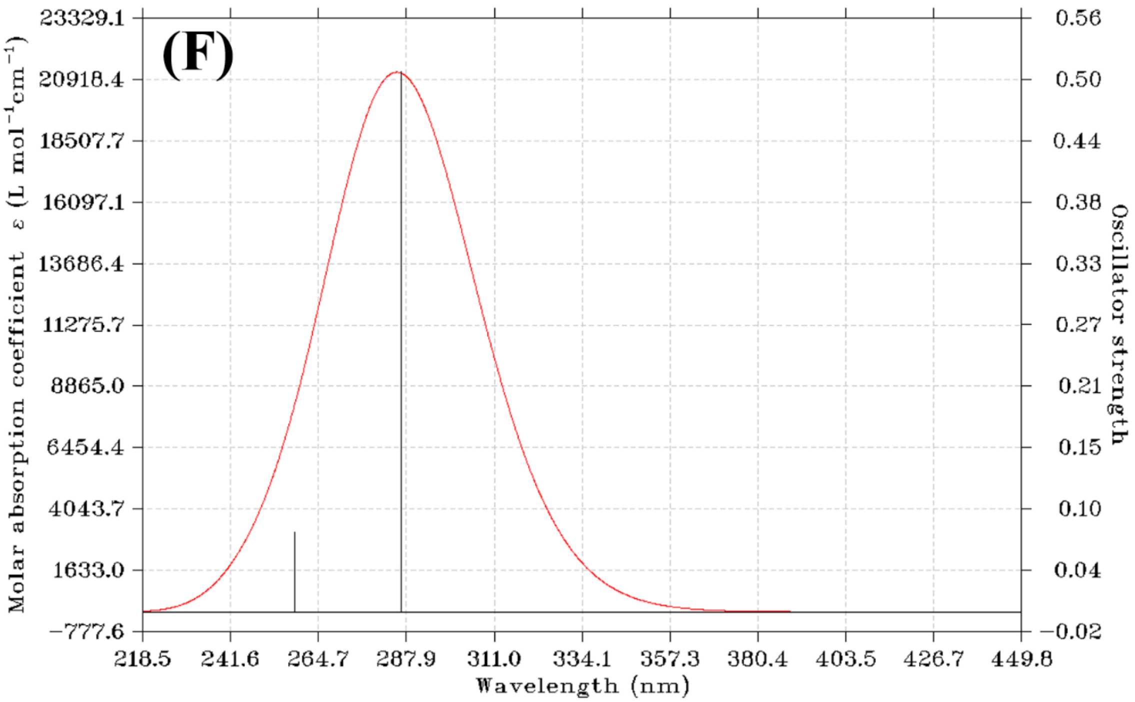

Figure S2. Simulated excitation (EX) spectra of **gefitinib** obtained from DFT calculations and visualized using **Multiwfn** software. Panels represent the predicted spectra in different media: **(A)** water, **(B)** MeCN, **(C)** Chloroform, **(D)** Methanol, **(E)** Ethanol, and **(F)** Gas phase.

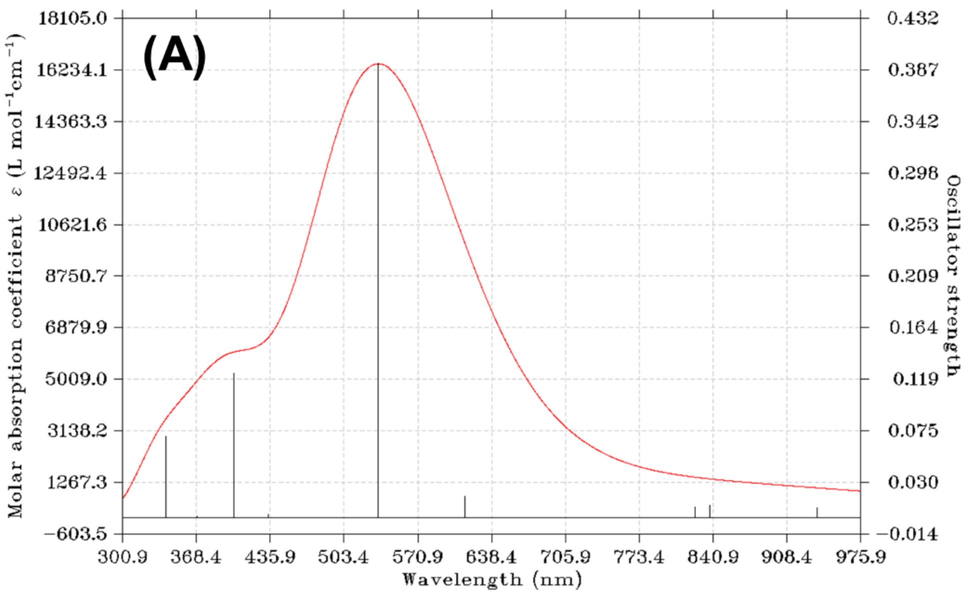
(A)
Molar absorption coefficient ε (L mol⁻¹cm⁻¹)
Oscillator strength
Wavelength (nm)
18105.0
16234.1
14363.3
12492.4
10621.6
8750.7
6879.9
5009.0
3138.2
1267.3
−603.5
0.432
0.387
0.342
0.298
0.253
0.209
0.164
0.119
0.075
0.030
−0.014
300.9
368.4
435.9
503.4
570.9
638.4
705.9
773.4
840.9
908.4
975.9

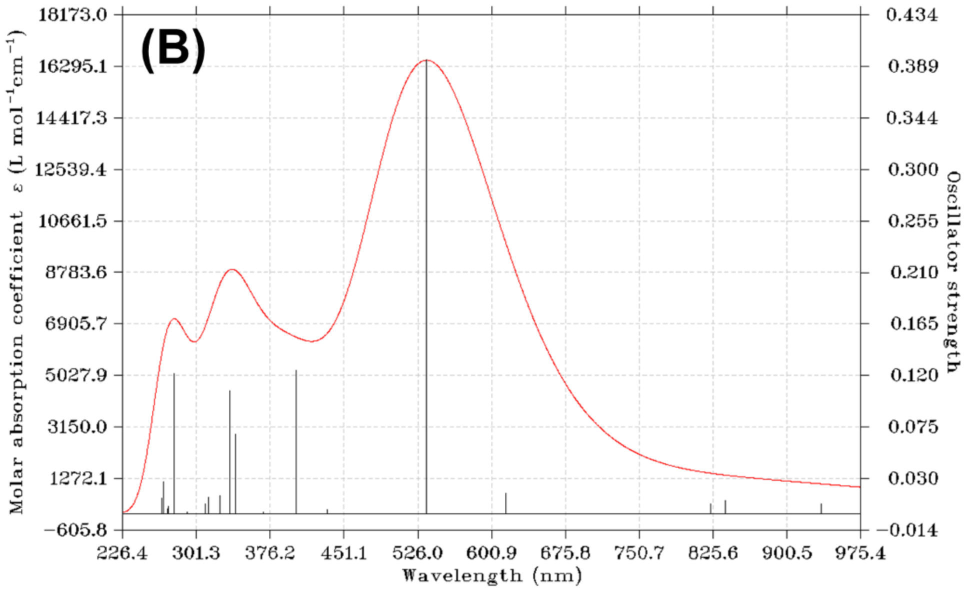
(B)
Molar absorption coefficient ε (L mol⁻¹cm⁻¹)
Oscillator strength
Wavelength (nm)
18173.0
16295.1
14417.3
12539.4
10661.5
8783.6
6905.7
5027.9
3150.0
1272.1
−605.8
0.434
0.389
0.344
0.300
0.255
0.210
0.165
0.120
0.075
0.030
−0.014
226.4
301.3
376.2
451.1
526.0
600.9
675.8
750.7
825.6
900.5
975.4

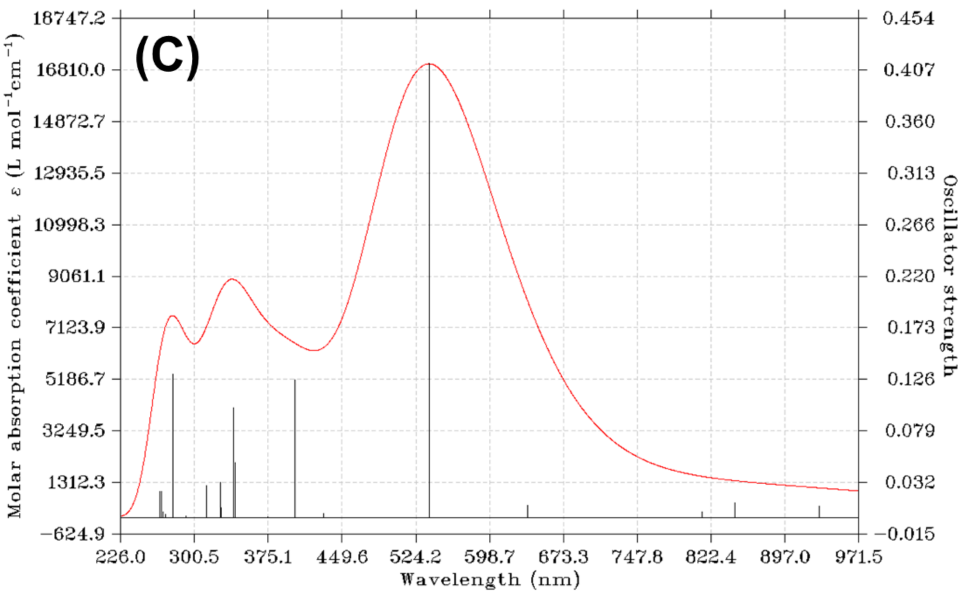
(C)
Molar absorption coefficient ε (L mol⁻¹cm⁻¹)
Oscillator strength
Wavelength (nm)
18747.2
16810.0
14872.7
12935.5
10998.3
9061.1
7123.9
5186.7
3249.5
1312.3
−624.9
0.454
0.407
0.360
0.313
0.266
0.220
0.173
0.126
0.079
0.032
−0.015
226.0
300.5
375.1
449.6
524.2
598.7
673.3
747.8
822.4
897.0
971.5

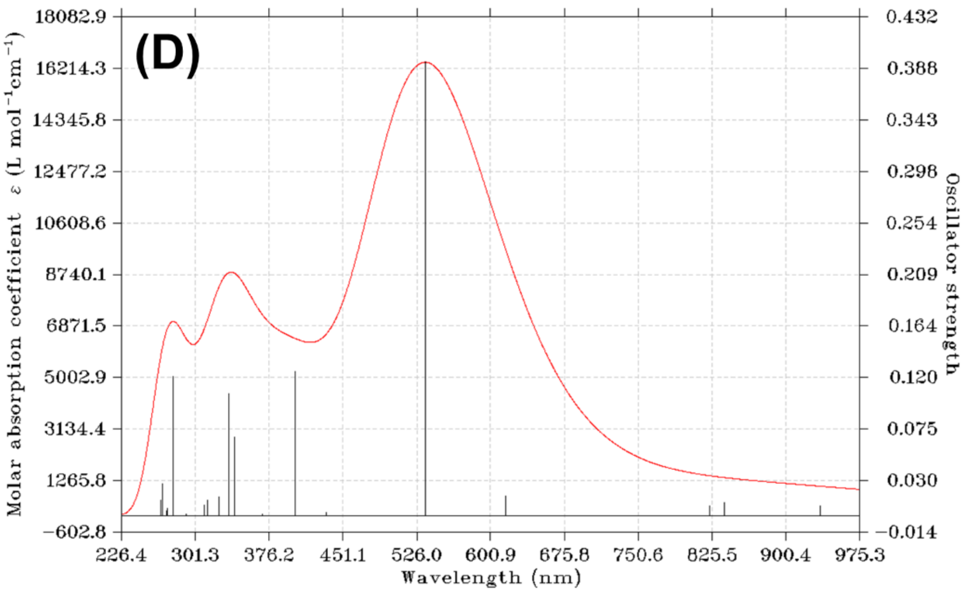
(D)
Molar absorption coefficient ε (L mol⁻¹cm⁻¹)
Oscillator strength
Wavelength (nm)
18082.9
16214.3
14345.8
12477.2
10608.6
8740.1
6871.5
5002.9
3134.4
1265.8
−602.8
0.432
0.388
0.343
0.298
0.254
0.209
0.164
0.120
0.075
0.030
−0.014
226.4
301.3
376.2
451.1
526.0
600.9
675.8
750.6
825.5
900.4
975.3

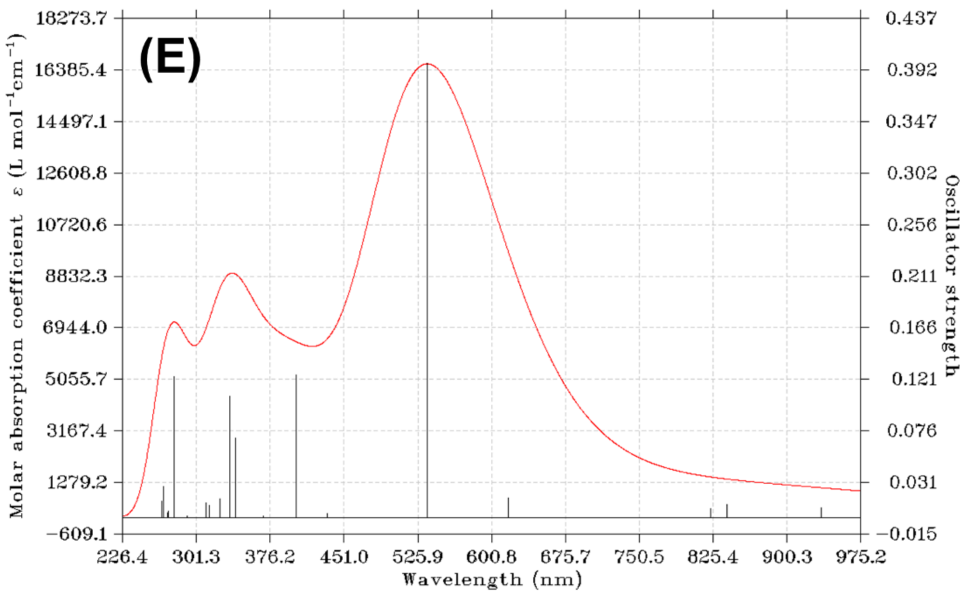

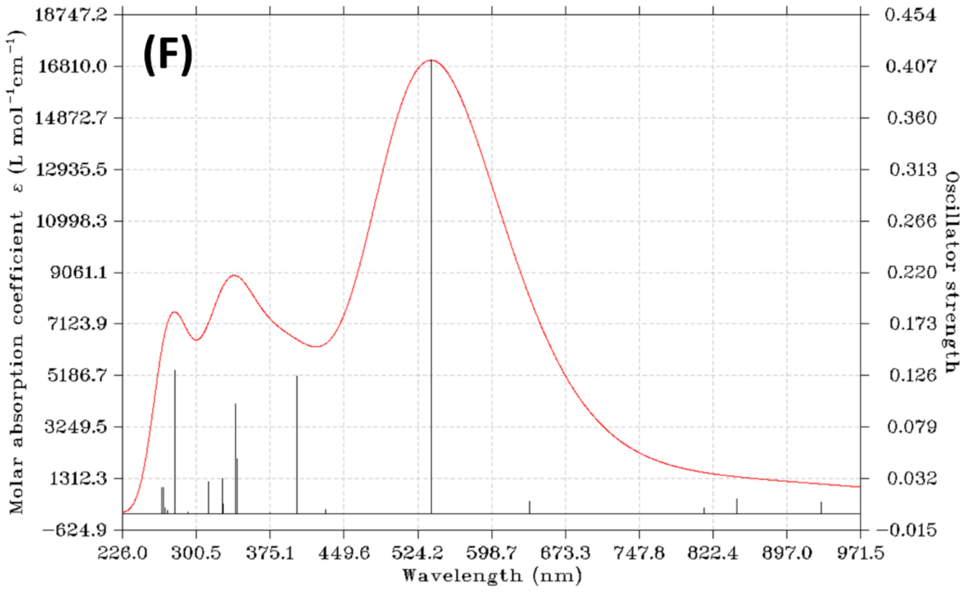

Figure S3. Simulated emission (EM) spectra of **gefitinib** obtained from DFT calculations and visualized using **Multiwfn** software. Panels represent the predicted spectra in different media: **(A)** water, **(B)** MeCN, **(C)** chloroform, **(D)** methanol, **(E)** ethanol, and **(F)** gas phase.
